# The Formation of the Bicoid Morphogen Gradient Requires Protein Movement from Anteriorly Localized mRNA

**DOI:** 10.1371/journal.pbio.1000596

**Published:** 2011-03-01

**Authors:** Shawn C. Little, Gašper Tkačik, Thomas B. Kneeland, Eric F. Wieschaus, Thomas Gregor

**Affiliations:** 1 Howard Hughes Medical Institute, Department of Molecular Biology, Princeton University, Princeton, New Jersey, United States of America; 2 Department of Physics and Astronomy, University of Pennsylvania, Philadelphia, Pennsylvania, United States of America; 3 Joseph Henry Laboratories of Physics, Princeton University, Princeton, New Jersey, United States of America; 4 Lewis-Sigler Institute for Integrative Genomics, Princeton University, Princeton, New Jersey, United States of America; New York University, United States of America

## Abstract

The Bicoid morphogen gradient directs the patterning of cell fates along the anterior-posterior axis of the syncytial *Drosophila* embryo and serves as a paradigm of morphogen-mediated patterning. The simplest models of gradient formation rely on constant protein synthesis and diffusion from anteriorly localized source mRNA, coupled with uniform protein degradation. However, currently such models cannot account for all known gradient characteristics. Recent work has proposed that *bicoid* mRNA spatial distribution is sufficient to produce the observed protein gradient, minimizing the role of protein transport. Here, we adapt a novel method of fluorescent in situ hybridization to quantify the global spatio-temporal dynamics of *bicoid* mRNA particles. We determine that >90% of all *bicoid* mRNA is continuously present within the anterior 20% of the embryo. *bicoid* mRNA distribution along the body axis remains nearly unchanged despite dynamic mRNA translocation from the embryo core to the cortex. To evaluate the impact of mRNA distribution on protein gradient dynamics, we provide detailed quantitative measurements of nuclear Bicoid levels during the formation of the protein gradient. We find that gradient establishment begins 45 minutes after fertilization and that the gradient requires about 50 minutes to reach peak levels. In numerical simulations of gradient formation, we find that incorporating the actual *bicoid* mRNA distribution yields a closer prediction of the observed protein dynamics compared to modeling protein production from a point source at the anterior pole. We conclude that the spatial distribution of *bicoid* mRNA contributes to, but cannot account for, protein gradient formation, and therefore that protein movement, either active or passive, is required for gradient formation.

## Abstract 2

New quantitative data show that the Bicoid morphogen gradient is generated from a dynamic localized source and that protein gradient formation requires protein movement along the anterior-posterior axis.

## Author Summary

The Bicoid protein gradient plays a crucial role in determining the anterior body pattern of *Drosophila* embryos. This gradient is the classic example of morphogen-mediated patterning of a developing metazoan and serves as a major topic for mathematical modeling. Accurate modeling of the gradient requires a detailed account of the underlying *bicoid* mRNA distribution. The classic model holds that mRNA protein gradient arises via protein diffusion from mRNA localized at the anterior of the developing egg. In contrast, recent proposals suggest that an mRNA gradient generates the protein gradient without protein movement. In this study, we introduce a novel mRNA quantification method for *Drosophila* embryos, which allows us to visualize each individual mRNA particle accurately in whole embryos. We demonstrate that all but a few mRNA particles are confined to the anterior 20% of the egg, and consequently that the protein must move in order to establish a gradient. We further report that the mRNA distribution is highly dynamic during the time of protein synthesis. In numerical simulations, we show that incorporating realistic spatial locations of the individual source mRNA molecules throughout the developmental period is necessary to accurately model the experimentally observed protein gradient dynamics.

## Introduction

Accurate development of metazoan embryos requires precise production, reception, and interpretation of patterning cues along appropriate spatial axes on realistic timescales. Many embryonic patterning events utilize graded spatial distributions of patterning molecules, or morphogens, whose activities rely fundamentally on molecular interactions susceptible to environmental fluctuations and stochasticity in gene expression [1]. Despite the widespread occurrence of morphogen-mediated tissue patterning, in most cases little or no quantitative data exist regarding the dynamics of gradient establishment or the spatio-temporal regulation of morphogen production.

The wealth of molecular and genetic tools available in *Drosophila melanogaster* offers an optimal context in which to study the basis of developmental accuracy in embryonic patterning by morphogens. In the *Drosophila* embryo, anterior-posterior (AP) axial patterning originates with maternal cues deposited into the developing egg [2]. Among these cues is the transcription factor Bicoid (Bcd), the mRNA of which localizes at the anterior cortex of the oocyte [3]–[6]. Translation of *bcd* mRNA is believed to commence upon fertilization, after which the embryo undergoes 13 rapid nuclear mitotic cycles (n.c.) without cytokinesis. By the start of interphase 14 about 2 h after egg deposition (AED), the embryo consists of a syncytial blastoderm layer of about 6,000 nuclei at the cortical surface, surrounding the interior core of yolk and vitellogenic nuclei. During the blastoderm stage Bcd protein distributes along the AP axis as an exponentially decaying gradient [7]–[9], and nuclear Bcd activates target genes in a dosage-dependent manner (reviewed in [10]).

Recently, quantitative analysis of living embryos expressing Bcd-GFP revealed that the protein gradient remains nearly unchanged subsequent to the arrival of nuclei to the cortex at n.c. 10 (about 85 min AED), and therefore that the nuclear gradient achieves stability within only about 80 min at 25°C [9]. Moreover, nuclear gradients at n.c. 14 exhibit remarkable reproducibility between embryos: along the AP axis, similarly positioned nuclei in different embryos contain Bcd-GFP concentrations that differ by only about 10% [9]. In principle, this level of precision would be sufficient for nuclei to distinguish their AP positions with an error of only a single nuclear diameter [11]. These observations raise fundamental questions regarding the underlying cell-biologic mechanisms responsible for the rapid, precise establishment of nearly equivalent gradients in essentially every embryo.

One such question concerns the dependence of protein gradient formation on the strength, localization, and dynamics of the underlying *bcd* mRNA. Females carrying altered genetic dosages of *bcd* produce gradients of altered amplitude, resulting in mispatterned embryos [12]–[14]. Moreover, the packaging of *bcd* mRNAs into discrete ribonucleoprotein (RNP) complexes, and their subsequent localization to the anterior oocyte cortex, requires a suite of maternal factors (reviewed in [15]–[17]). Ooctyes lacking these factors produce embryos with distorted protein gradients resulting from *bcd* mRNA translation at inappropriately posterior locations [12]. The reliable formation of the Bcd protein gradient must depend, at least in part, on the spatial distribution and the number of *bcd* mRNA molecules. While prior work has utilized in situ hybridization to document the spatial distribution of *bcd* mRNA during embryogenesis [3]–[5],[18],[19], no work has yet determined *bcd* mRNA particle numbers or examined their global localization in the developing embryo.

A second question regards the biophysical processes affecting the Bcd protein, such as its transport and degradation (reviewed in [20]). The observed exponentially decaying nuclear steady state Bcd protein distribution is consistent with analytical models of gradient formation via constant anterior protein synthesis, coupled with diffusion and uniform degradation throughout the embryo (the SDD model). Within this model, the effective diffusion constant of the Bcd protein D and the protein lifetime τ determine the dynamics of gradient formation. A relatively short τ would allow the gradient to reach an equilibrium distribution (i.e. when protein degradation is matched by new synthesis) within the available developmental time of ∼2 h. Conversely, a relatively long protein lifetime will prohibit the achievement of such an equilibrated state within that time. Likewise, larger or smaller values of D will increase or decrease, respectively, the predicted distance a protein can move away from its source mRNA. A direct measurement of D for cytoplasmic Bcd-GFP at n.c. 13 [9] yielded a substantially slower diffusion constant than for comparably sized, biologically inert molecules [21], and about an order of magnitude too low to account for the rapid achievement of a steady state gradient with the appropriate length constant. More recent work has suggested the presence of multiple populations of Bcd-GFP, where a fraction of Bcd-GFP may diffuse rapidly enough to establish stability in the time available [22],[23].

Slow diffusivity, however, would be consistent with an alternate model in which the protein gradient arises from graded *bcd* mRNA distribution [19]. Low values of D coupled with rapid protein degradation would minimize protein movement away from source molecules, so that the local mRNA amount would predominately determine protein concentration along the AP axis. Under these conditions, sufficiently mobile mRNA would greatly impact the dynamics of gradient formation, and protein diffusion contributes only minimally. However, at present it is difficult to evaluate the legitimacy of these models, or to determine what values of D and τ, if any, might accurately describe the dynamics of Bcd gradient formation, because no quantitative data exist which span the first 80–90 min of development.

To address these questions, we developed two novel quantitative measurement approaches. First, we adapted a method of fluorescent in situ hybridization to the *Drosophila* embryo employing fluorescently labeled DNA oligonucleotides [24],[25]. This method allowed us to identify individual *bcd* mRNA particles and determine their positions and intensities. We found that *bcd* mRNA particles undergo movement into the core of the embryo, away from their initial site of localization at the ooctye cortex, by the end of the third cleavage division (within 30 min AED). Subsequently, *bcd* mRNA is relocalized to the egg cortex during cortical nuclear migration (mitoses 6 to 9, 55 to 75 min AED), during which *bcd* particles tend to dissociate without *bcd* mRNA degradation. Despite the dynamic behavior of *bcd* particles, at all times >90% of mRNA is localized within the anterior 20% of the embryo, a distribution which is insufficiently extended to produce the observed protein gradient in the absence of large-scale protein redistribution along the anterior-posterior axis.

Second, we measured nuclear Bcd-GFP levels during presyncytial stages using GFP fluorescence in fixed embryos. We discovered that Bcd-GFP does not begin accumulating in nuclei until interphase 6, about 45 min AED, reaching peak levels 50 min later in n.c. 11 and 12. Unexpectedly, in fixed samples we found that the nuclear gradient declines between n.c. 13 and n.c. 14, a result which differs from prior analysis of living embryos. We present evidence that attributes this difference in part to delayed fluorescence maturation of GFP in living embryos.

Finally, to examine whether the *bcd* mRNA distribution impacts protein gradient formation, we incorporated its observed spatio-temporal dynamics into numerical simulations of protein production and movement. We found that models utilizing the actual mRNA distribution result in improved predictions of protein gradient dynamics, compared to models employing a single point source at the anterior pole. Therefore, we conclude that although the spatially extended mRNA localization contributes to the protein gradient, mRNA localization alone cannot account for protein gradient dynamics. Our results demonstrate that protein movement, whether active or passive, is a necessary component of Bcd protein gradient formation.

## Results

### Fluorescently Labeled DNA Oligonucleotides Allow Visualization of Individual Diffraction-Limited *bcd* RNP Complexes

**Figure 1:**
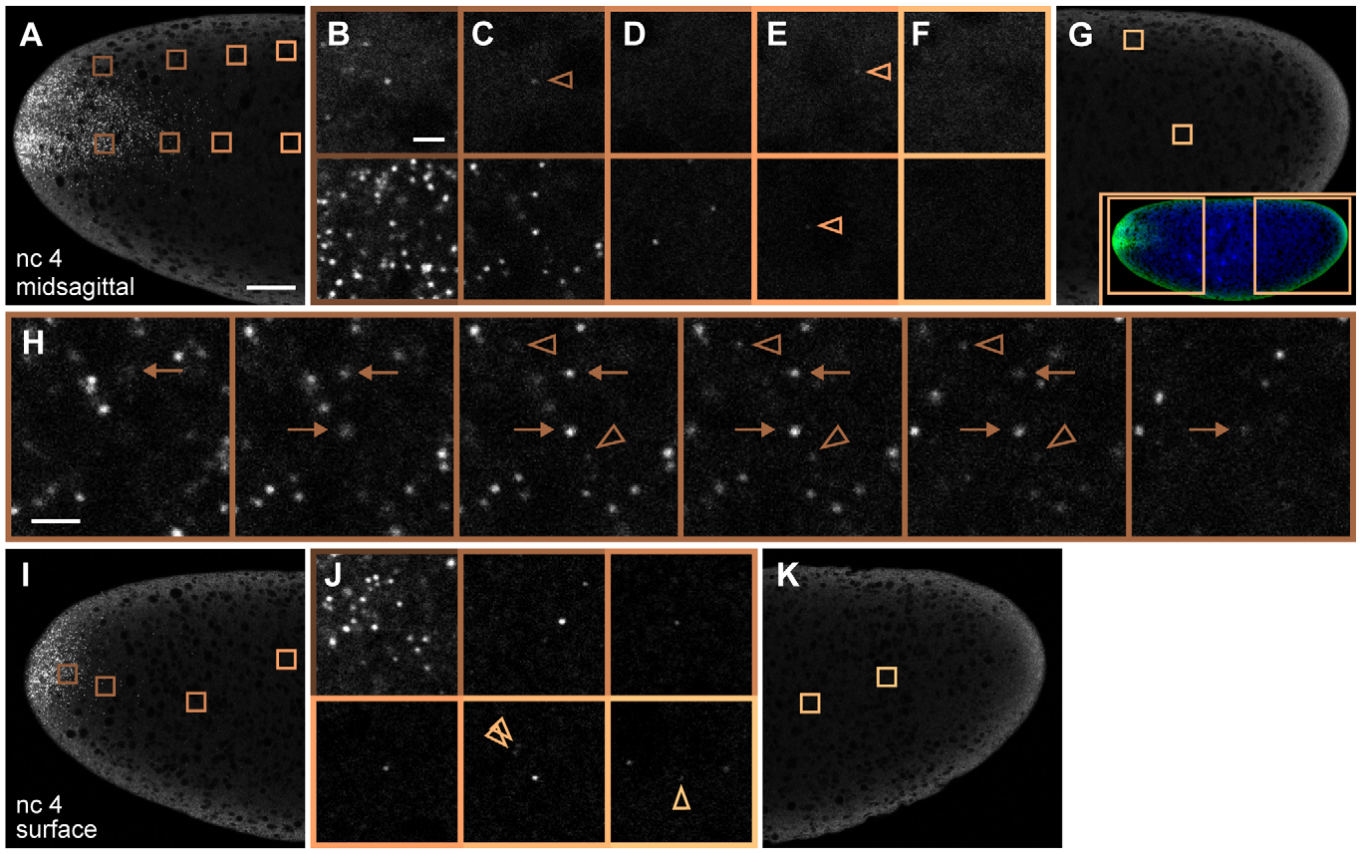
Visualization of discrete *bcd* mRNA particles with fluorescently labeled oligonucleotides. (A–G) Confocal slices through the midsagittal plane at the anterior (A) and posterior (G) of a wild-type embryo during nuclear cycle (n.c.) 4. Copper-shaded boxes in (A) and (G) indicate regions shown in magnified views (B–F); top row, near embryo surface; lower row, center views; arrowheads indicate selected dimly fluorescent particles. (G inset) Low magnification image of whole embryo (mRNA in green, DNA in blue), boxed regions are shown in (A) and (G). (H) Six sequential confocal *z*-slices beginning 0.8 µm below (leftmost panel) and ending 1.2 µm above (rightmost panel) the image shown in (C); lower panel in (C) is identical to third panel from the left in (H). Arrows: bright particles appearing on five slices. Arrowheads: weak particles appearing on three slices. (I–K) Confocal slices of the same embryo imaged ∼5 µm beneath cortical surface. (J) Magnified views of selected regions indicated by six shaded boxes in (I) and (K). Here and in Figure 2, posterior regions containing particles were deliberately chosen for zoomed views; however, the majority of the posterior does not contain detectable particles. Scale bars: 25 µm (A), 2 µm (B, H).

**Figure 2:**
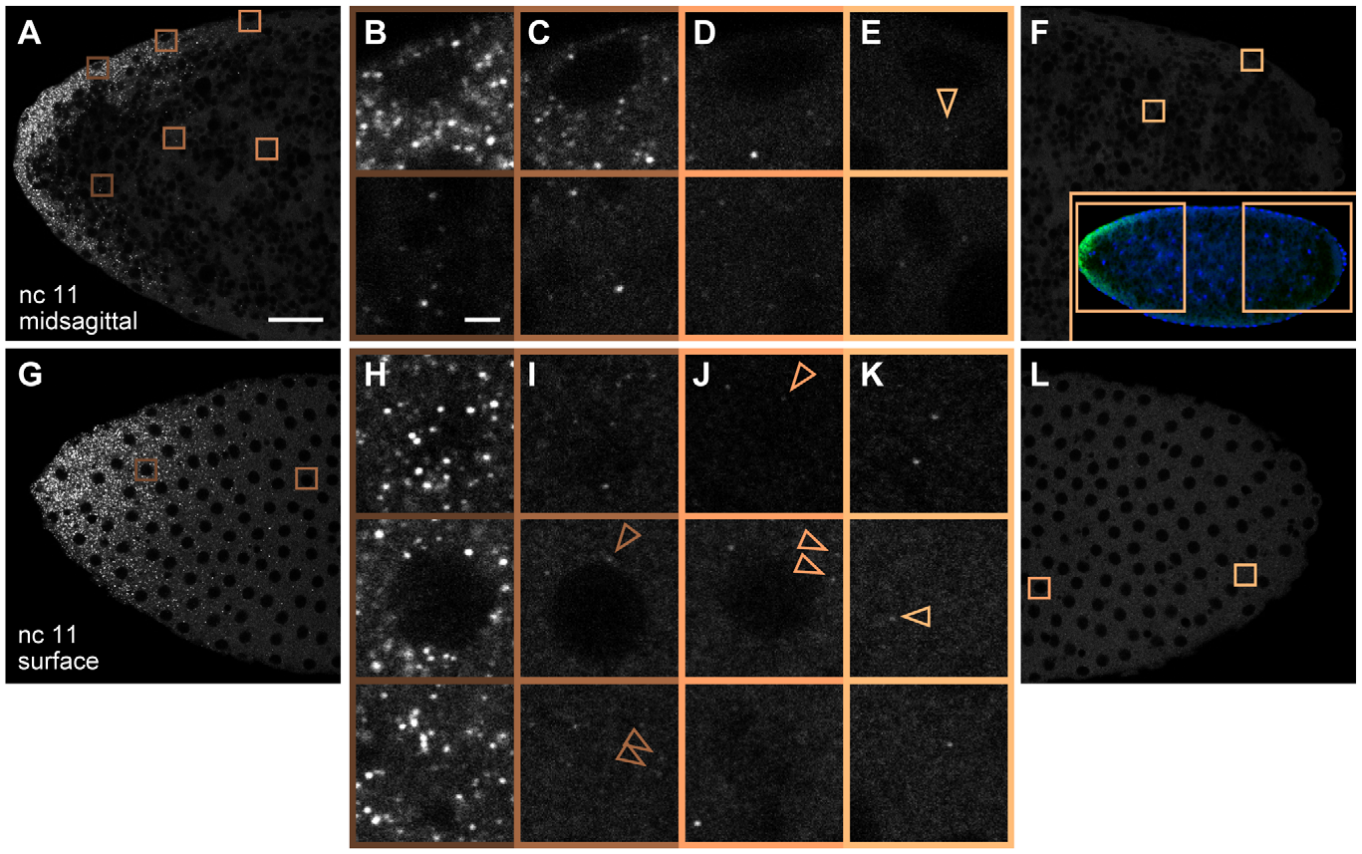
*bcd* mRNA particles surrounding surface nuclei during syncytial blastoderm stages. (A–F) n.c. 11 embryo near the midsagittal plane at the anterior (A) and posterior (F). Shaded boxes indicate magnified views (B–E) corresponding to cortex (upper) or core (lower); arrowheads indicate selected faint particles. (F inset) Low magnification image of whole embryo (mRNA in green, DNA in blue), boxes indicate regions shown in (A) and (F). (G–L) Same embryo imaged at the nuclear layer. (H–K) Selected *z*-slices of boxed regions indicated in (G) and (L) showing apical (upper panels) and basal (lower panels) planes surrounding the nuclear layer (middle panels). Arrowheads indicate selected dimly fluorescent particles. Scale bars: 25 µm (A), 2 µm (B).

To examine *bcd* mRNA distribution in fixed embryos, we adapted a fluorescent in situ hybridization (FISH) protocol [24] using a set of 48 20-mer DNA oligonucleotides complementary to the *bcd* open reading frame (Table S1) directly conjugated to AlexaFluor fluorophores [25]. This approach has been used previously to detect single mRNA molecules in mammalian cell culture and *C. elegans* embryos [25]. By employing directly labeled oligonucleotides as probes, we bypassed the use of antibodies and enzymes for detection [26]–[28], and thus minimized nonspecific background, nonlinear signal response, variable tissue penetration by reagents, or other potential difficulties associated with quantification of conventional FISH signal [29]. Using standard laser scanning confocal microscopy, we can readily detect the anterior localization of *bcd* mRNA at low magnification (Figure S1) and distinguish individual *bcd* RNP complexes in high resolution images (Figures 1 and 2).

To obtain the complete three-dimensional (3d) structure of *bcd* mRNA distribution, we generated high resolution confocal stacks at high magnification, such that a spatial unit voxel represents a volume of 75 nm×75 nm×420 nm. These stacks span the entire left or right half of each embryo from the midsagittal plane to the embryo surface, representative of the entire embryo due to left-right symmetry. Figures 1 and 2 show typical images of embryos labeled during interphase 4 and interphase 11, respectively. Corresponding 3d stacks are provided in Movies S1 and S2. Images taken at the anterior of labeled embryos reveal readily identifiable individual *bcd* RNP particles above background noise (Figures 1A–E and 2A–D), whereas the embryo posterior is essentially devoid of *bcd* mRNA particles (Figures 1F–G and 2E–F). Individual particles appear on multiple neighboring z-slices (Figure 1H), owing to the objective's spatially extended point spread function (PSF).

**Figure 3:**
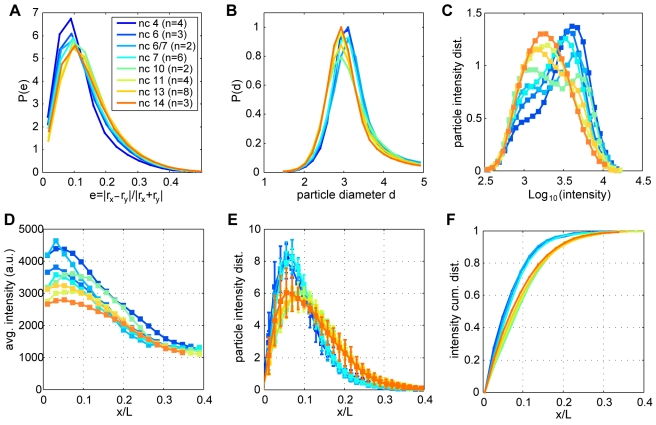
Quantification of diffraction-limited *bcd* mRNA particles. Individual particles identified in anterior stacks of 32 embryos hybridized with fluorescently labeled *bcd* oligonucleotides ranging from n.c. 4 to n.c. 14 (see color code in legend). (A) *bcd* particle eccentricity *e* calculated as the ratio of the difference of the major and minor particle axes (r_x_ and r_y_) to their sum; *e* = 0 and *e* = 0.3 correspond to r_x_/r_y_ = 1 (perfectly circular) and r_x_/r_y_ = 2, respectively. Shown is the probability distribution of *e* across all identified particles. (B) Probability distribution of *bcd* particle diameters d with a mean of 3.05±0.10 pxl and a standard deviation of 0.42±0.05 pxl (1 pixel = 75.7 nm). (C) Distribution of *bcd* particle intensities (note log units on *x*-axis). (D) Average *bcd* particle intensity as a function of distance along the AP axis in fractional egg length x/L. (E) Distribution of fluorescence intensities in *bcd* mRNA particles along the AP axis; error bars are standard deviations across embryos (note difference from total *bcd* particle distribution in Figure S4). (F) Cumulative distribution of *bcd* mRNA fluorescence intensity (cumulative plot of (E)).

To obtain counts of discrete *bcd* mRNA particles, their localization, and fluorescence intensities, we designed custom image analysis software to determine the spatial positions, radii, and fluorescence intensities of *bcd* mRNA particles (see Materials and Methods). As illustrated in Figure S2, the analysis algorithm discriminates between overlapping particles which densely populate the anterior and readily detects dim particles. A large majority of detected *bcd* particles are circular in shape (Figure 3A), confirming that the algorithm resolves closely neighboring particles. The particles have a spatial extent of about 3 pixels on average, corresponding to a physical distance of ∼200 nm (Figure 3B). This is identical to the PSF in the confocal slices (Figure S3), indicating that the particles are smaller than the diffraction limit of our microscopy. The extended PSF dictates that each mRNA particle must be detected simultaneously on consecutive z-slices, and the algorithm uses this property to discriminate true particles from local background noise (see Materials and Methods). We identified an optimal threshold for distinguishing between candidate particles and random fluorescence fluctuations by examining posterior stacks which contain few particles (see Materials and Methods and Figure S4). As additional controls, we examined background fluorescence in cleavage stage embryos processed without any probes (Figure S5A–C) or exposed to probes against the purely zygotically expressed gene *giant* (Figure S5D–F). Both show a low level of fluorescent signal, but no detected particles. These controls allow us to exclude falsely identified particles.

*bcd* particles coalesce in vivo during *bcd* mRNA anterior localization in late oogenesis, and subsequently disperse upon egg activation [18],[30],[31], suggesting that *bcd* RNPs may remain at least partially intact in fertilized embryos. The fluorescent intensities of particles observed by our method span about a 10-fold range (Figure 3C). This broad distribution suggests the presence of multiple *bcd* mRNAs in a single detected particle, consistent with previous biochemical and fluorescence microscopy observations [32]–[34]. *bcd* mRNA is not degraded until the onset of cellularization at n.c. 14 [3]. Therefore, it is highly likely that the broad intensity distribution reflects variation in the number of RNAs per particle. Fluorescence variability is reflected in the axial (z) diameter of particles: we observe that bright particles occupy up to 5 z-slices, whereas dim particles are detectable above noise level in fewer slices (Figure 1H). Our ability to distinguish discrete particles affords an unprecedented quantitative view of mRNA particles and allows us to separate their dynamics from the ultimate dynamics of Bcd protein.

### *bcd* RNP Complexes Form a Short Anterior Gradient at All Times, With <1% of *bcd* Present in the Posterior 60% of the Egg

To quantify the spatial distributions of *bcd* mRNA, we examined embryos ranging from n.c. 3 to n.c. 14. During all stages of development, *bcd* particles are found in a graded distribution peaking at around 7% egg length (EL, as measured from the anterior pole) and leveling off at nearly zero particle density by 40% EL (Figure S6). Confocal images taken at both early cleavage (Figure 1) and blastoderm (n.c. 11, Figure 2, and n.c. 13, Figure S7) stages reveal a higher density of *bcd* particles in anterior compared to posterior regions. Moreover, anteriorly localized particles are brighter than those toward the posterior (Figure 3D). The higher density of brighter anterior particles results in a sharp, steep gradient of total *bcd* mRNA distribution within the anterior third of the embryo (Figure 3E). At all times, 90% or more of total *bcd* mRNA is found within the anterior 20% of the embryo (Figure 3F), as previously reported [35]. The quantitative difference between Figures 3E and S4A arises from both the increased number and intensity of anterior particles.

Although the majority of mRNA particles are detected in the anterior 20%, we nevertheless observe faint discrete particles scattered along the entire AP axis. During both cleavage and blastoderm, very few or no particles are detected in the posterior embryo core (Figures 1F–G, 2E–F, S7E–F). Conversely, a small number of particles exhibiting weak fluorescence sparsely populate the posterior surface (Figures 1I–K, 2G–L, S7I–L). A handful of particles can be detected even at almost 100% EL near the posterior pole (Figure 2K–L). Thus, we can document even the small fraction of particles that either fail to localize during oogenesis or which are transported an unusually large distance away from the anterior cortex after egg activation. To our knowledge, *bcd* RNP complexes have not been detected in the posterior of wild-type embryos. These observations demonstrate the exquisite sensitivity of our method to the presence of dimly fluorescent *bcd* mRNA particles. However, we emphasize that the particles found in the entire posterior 60% of the embryo constitute less than 1% of the total *bcd* mRNA (Figure 3F). These particles are uniformly dimly fluorescent (Figure 3D) and are found at a sparse density which does not change significantly during development. Translation of *bcd* mRNA is repressed by Nanos activity [36], silencing posteriorly localized *bcd* mRNA. Based on these observations, the fraction of *bcd* mRNA present in the posterior 60% of the embryo likely contributes only negligibly to protein gradient formation.

### *bcd* RNP Complexes Translocate from the Embryo Core to the Cortex Concomitant with Nuclear Expansion

Previous studies have demonstrated that egg activation triggers release of *bcd* mRNA from its initial tight localization at the anterior egg cortex, resulting in a posterior dispersion of *bcd* mRNA within 25 min at 25°C [30]. This corresponds to the interphase of n.c. 3 (Figure S8), by which time *bcd* mRNA has already reached its most posterior extent, where it remains from n.c. 4 through 6 (Figure 3E and 3F). The AP particle distribution at n.c. 4 therefore results from this early release of *bcd* mRNA from the cortex. Prior to and during n.c. 6, 97% of all *bcd* mRNA is found in the anterior 20% (Figure 3F), with the remainder forming a gradient which drops to nearly zero before 40% EL.

In midsagittal planes at n.c. 4, regions near the embryo surface contain fewer particles than the center of the embryo (compare upper and lower panels in Figure 1B–D). Consistent with this, confocal planes taken near the embryo surface contain fewer particles compared to the same AP position at the midsagittal plane (compare Figure 1A and 1I). Therefore, much of *bcd* mRNA is not near the cortex but is found in the embryo interior (Figures 1A, S1A–B, S8), as observed previously [5]. In low magnification images, we often observe mRNA localization in a wedge or cone-shaped distribution jutting in toward the interior core of the embryo in midsagittal planes (Figures S8, S9A–D), suggesting the presence of uncharacterized structures along which *bcd* mRNA particles might translocate upon egg activation. The observation that particles do not progress further into the posterior after n.c. 3 supports the idea that *bcd* mRNA is tethered to underlying cytoskeletal structure(s) throughout embryogenesis [32] and is not free to diffuse.

**Figure 4:**
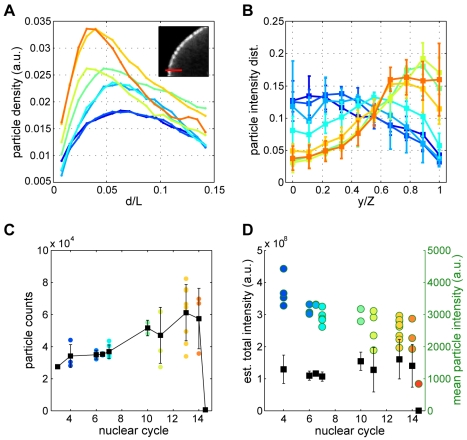
*bcd* mRNA particle dynamics, disassembly, and stability. All panels are generated on same data set as in Figure 3, with identical color labeling for different nuclear cycles. (A) *bcd* particle density as a function of fractional distance from the cortex d/L (d is the red line in inset of partial image of DAPI stained embryo) in a single midsagittal plane. (B) Probability distribution of bcd particle intensities along the z-axis within a confocal stack (error bars are standard deviations across embryos). (C) Absolute number of *bcd* particles as a function of n.c. Each dot corresponds to total number count in full anterior stack of an individual embryo (stack encompasses left or right embryo-half; due to left-right symmetry, the full embryo contains twice the number of particles indicated). Black squares and error bars correspond to averages and standard deviations across embryos at a given n.c., respectively (black line is a guide to the eye). Note the sharp drop during n.c. 14 (time point corresponds to 15 min after the end of mitosis 13; the actual average particle count below 50). (D) Average total *bcd* particle intensity as a function of n.c. (black squares; error bars are standard deviations across embryos in a given n.c.). In color, for individual embryos, mean *bcd* particle intensity as a function of n.c. (green scale on right).

We observed a marked change in *bcd* mRNA spatial localization after interphase 6. As the nuclei undergo expansion toward the axial poles beginning at the sixth mitosis and continuing at n.c. 7, *bcd* mRNA moves ahead of the expanding nuclei (Figures 4A, S1C–E). *bcd* mRNA progresses to the cortex during nuclear cortical migration between n.c. 8 to 10, such that in midsagittal planes at n.c. 11, *bcd* mRNA is highly enriched near the embryo cortex, with the majority of fluorescence found within about 25 µm of the embryo surface (Figures 2A–G, 4A). This enrichment is also apparent in z-slices collected at the cortical nuclear layer (Figures 2G–L, 4B, S7G–L). Compared to earlier cycles, bright *bcd* particles are now present on the surface at a greater distance from the anterior pole (compare Figure 1I to Figure 2G). Thus, with the nuclei penetrating the cloud of *bcd* mRNA during their cortical migration, by the start of the syncytial blastoderm stage, *bcd* mRNA fluorescence resembles a cup covering the anterior end of the embryo (Figure S9E–H), which remains in place through n.c. 14 (Figures 3E, 3F, S1F–G, and S6).

Concomitant with *bcd* particle translocation from core to cortex, we observe a mild posterior shift of the *bcd* mRNA distribution along the AP axis by the onset of the blastoderm stage at n.c. 10 (Figures 3E, 3F, and S6), as previously observed [5]. Embryos at all blastoderm stages appear similar, with *bcd* mRNA particles surrounding the nuclei on both the basal and apical surfaces (compare n.c. 11 in Figure 2 and n.c. 13 in Figure S7). Despite dynamic cortical relocalization, more than 90% of *bcd* is found in the anterior 20% of the embryo (Figure 3F), with the remaining 10% falling to zero particle density by 40% EL.

### *bcd* mRNA Particles Disassemble During Cortical Translocation, But *bcd* mRNA Is Not Degraded Until the Onset of Cellularization

Concomitant with the particle relocalization, the distribution of fluorescence intensities shifts from bright to dim spot intensities (Figure 3C), and the number of detected particles tends to increase with developmental age from about 70,000 per embryo in cycles prior to n.c. 7, to about 110,000 particles in blastodermal stages (Figure 4C). Despite these changes, the total fluorescent signal from *bcd* mRNA remains constant across all developmental times through early n.c. 14 (Figure 4D), arguing that our method is capable of detecting the constant maternal *bcd* mRNA pool. Given previous work indicating the presence of multiple *bcd* mRNA molecules per particle [32]–[34], these observations indicate that such particles disassemble during their relocalization to the cortex and suggest that mRNA degradation likely plays little role in particle dissolution.

**Figure 5:**
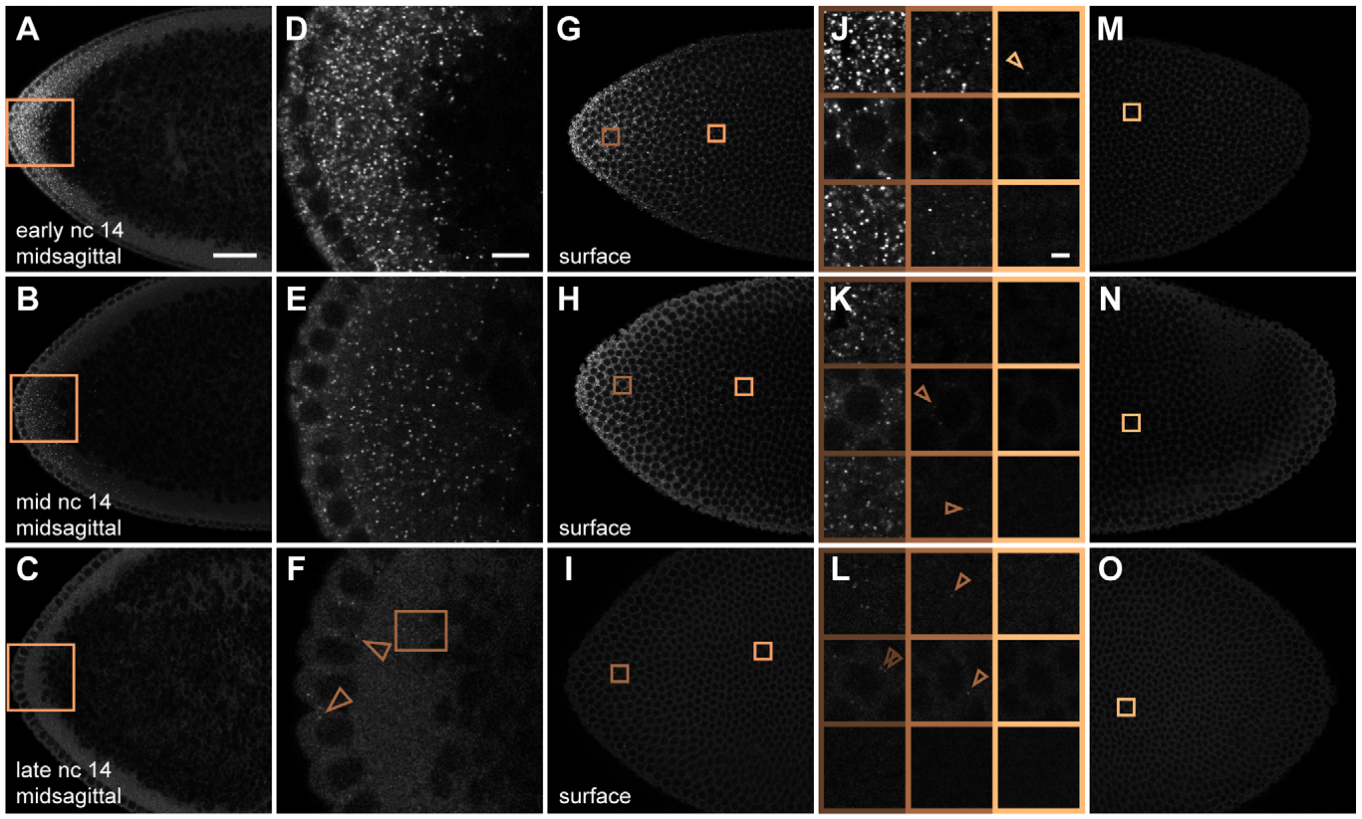
Uniform *bcd* mRNA particle degradation at the onset of cellularization. Each row shows an embryo during early (0–5 min, top row), mid- (5–10 min, middle row), or late (10–20 min, bottom row) n.c. 14 (times are approximate after mitosis 13). (A–C) Anterior midsagittal views. Boxed regions are magnified in (D–F). (G–O) Images of nuclear layer at embryo cortex; (G–I), anterior; (M–O), posterior; (J–L) magnified views of boxed regions apical (top panels) and basal (bottom panels) to nuclei (middle panels). Arrowheads indicate selected faint particles. Box in (F) highlights a cluster of particles. Scale bars: 25 µm (A), 5 µm (B), and 2 µm (J).

*bcd* RNA is degraded with other maternal mRNAs at the midblastula transition during n.c. 14; prior to this time, no degradation of *bcd* mRNA occurs [4],[37]. To contrast *bcd* mRNA particle disassembly with mRNA degradation, we documented the loss of *bcd* mRNA during n.c. 14 (Figure 5), which spans about 1 h at 25°C. During this time, wholesale zygotic gene expression is activated, maternal mRNAs are degraded, and cellular membranes form between the cortically positioned nuclei. We gauged the approximate age of fixed embryos in n.c. 14 on the presence of the cellularization front and the morphology of nuclei which elongate during cellularization. Immediately following the 13^th^ mitosis, embryos exhibit particles with intensity and spatial distributions similar to the earlier blastoderm stages (Figure 5A, 5D, 5G). During the next ∼10 min, the cell surface protrudes above each nucleus and membrane invagination begins [38],[39], during which we observe a dramatic reduction in overall fluorescence and a sharp decrease in the number of particles and fluorescence of particles in all regions of the embryo (Figure 5B, 5E, 5H, 5J–L). As nuclei undergo elongation, *bcd* mRNA is almost completely lost, and we detect only a handful of dim particles in the anterior, usually at the cortical or lateral surfaces between nuclei (Figure 5F, 5L). At this time, the total particle count has dropped to essentially zero (see the final data point on *x*-axis in Figure 4D). Thus, degradation of *bcd* can be readily distinguished from the separate earlier event of particle dissolution.

In summary, we have observed that *bcd* mRNPs behave dynamically in the embryo: after fertilization, *bcd* mRNA establishes a wedge-shaped distribution, which is subsequently reformed into a cup-shaped geometry during n.c. 6–10 (Figure S9), followed by mRNA decay at the onset of cellularization. Despite these dynamic rearrangements, more than 99% of all mRNA particles occupy the anterior 40% of the egg at all times. These findings demonstrate that an exponentially graded protein distribution, which is detectable as far as the posterior 85% of the egg [8],[9],[40], can be established only if the protein moves away from the anterior source.

### Nuclei Begin Accumulating Bcd Within 45 Min of Egg Deposition

**Figure 6:**
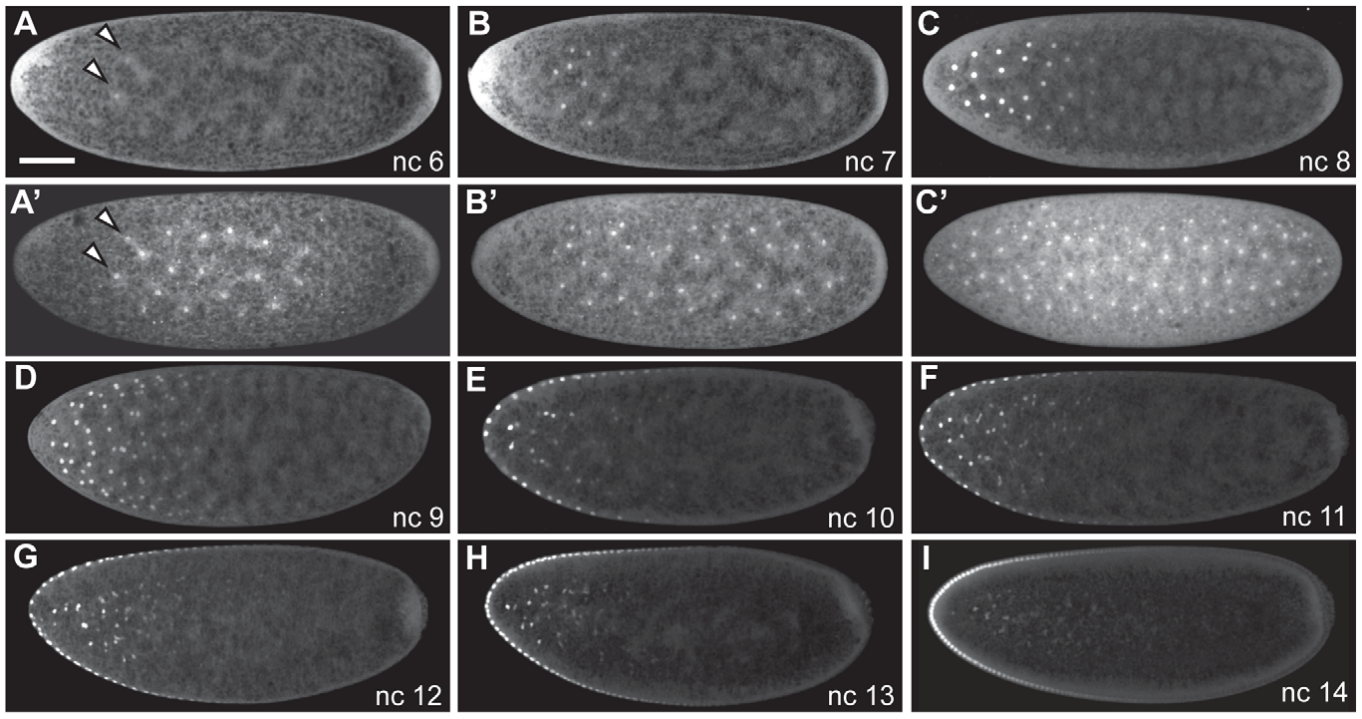
Bicoid gradient formation. Presyncytial blastoderm stage (A–D) and syncytial stage (E–I) embryos expressing Bcd-GFP. Images are maximum-projections of confocal stacks to display nuclei residing in several focal planes (A–D) or midsagittal confocal slices (E–I, 7 µm thick sections). Nuclear cycle (nc) is indicated. DAPI staining shown (A′–C′) to indicate positions of nuclei. Scale bar: 50 µm. Arrowheads in (A) show nuclear Bcd-GFP in n.c. 6, i.e. the earliest nuclear Bcd-GFP detected in any examined embryo.

Understanding the degree to which a graded mRNA source influences the formation of the Bcd protein gradient requires a comparison of mRNA and protein dynamics. Live imaging of transgenic embryos suggests that nuclear Bcd-GFP levels approach a nearly stable concentration gradient during the 10^th^ interphase, approximately 80–90 min AED [9]. Gradient formation must occur prior to this time; however, no previous study has quantified Bcd distributions in living cleavage stage embryos, due to challenges to optical measurements created by the greater opacity and autofluorescence of presyncytial embryos. To minimize these complications, we devised a specific fixation protocol (see Materials and Methods), which preserves GFP fluorescence of Bcd-GFP expressing embryos, allowing us to detect and measure nuclear Bcd-GFP with conventional confocal microscopy (Figure 6).

The earliest time at which we can detect nuclear Bcd-GFP is about 45 min AED, when Bcd-GFP begins to accumulate in the most anteriorly positioned nuclei during the first 2 min of the sixth interphase (Figure 6A), coinciding with the expansion of nuclei into the cloud of *bcd* mRNA and with the onset of *bcd* mRNA relocalization. We detect Bcd-GFP fluorescence above background autofluorescence in nuclei positioned within the anterior third of the embryo when the nuclei extend from about 25% to 60% EL (Figure 6A). In contrast, during interphase 5 nuclear Bcd-GFP appeared no greater than background (Figure S10; *n* = 12 embryos), showing that Bcd-GFP has not accumulated to high enough levels for visualization in nuclei. Tissue autofluorescence may preclude detection of lower levels of nuclear localized Bcd-GFP; therefore, Bcd-GFP accumulation begins during n.c. 6 at the latest. Delayed nuclear accumulation would be consistent with previous work suggesting that the translation rate of *bcd* may be relatively low during the first hour of development before polyadenylation of *bcd* mRNA [41].

### The Nuclear Bcd Gradient Forms During n.c. 6–10, Is Stable During n.c. 11–13, and Decays Thereafter

Between the sixth mitosis and interphase 10, nuclei migrate toward the egg cortex as an expanding, elliptical shell, while continuing to accumulate Bcd-GFP (Figure 6A–D). A gradient of nuclear Bcd-GFP can be readily distinguished within the anterior third of the embryo from n.c. 7 and 8 (Figure 6B, C). By n.c. 9, Bcd-GFP is apparent in nuclei throughout the anterior half of the embryo (Figure 6D), demonstrating the continued heightening of Bcd-GFP levels along the AP axis. The nuclei arrive at the egg cortex and form the syncytial blastoderm upon completing the ninth mitosis. From n.c. 10 onward, the Bcd-GFP gradient is evident in single confocal slices of the midsagittal plane (Figure 6E–I), reminiscent of images of Bcd-GFP obtained previously by live imaging [9].

**Figure 7:**
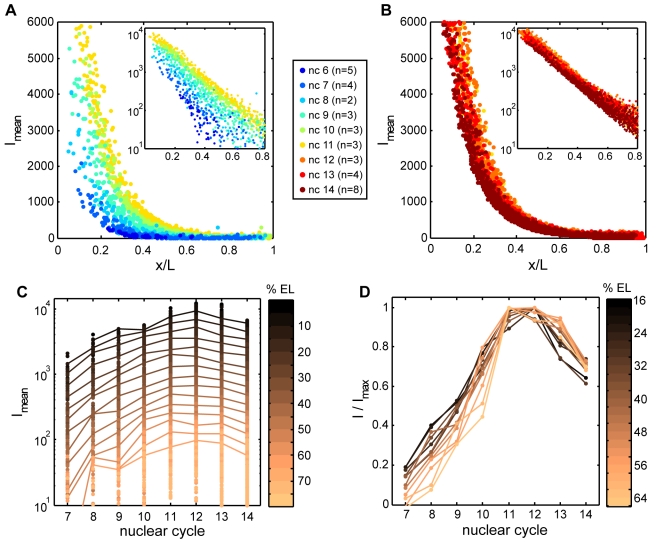
Bcd gradient dynamics. (A–B) Mean attenuation-corrected nuclear Bcd-GFP gradients as a function of relative position along the AP axis extracted from 35 embryos expressing Bcd-GFP fixed in n.c. 6 to n.c. 14; each dot corresponds to an individual nucleus (see Materials and Methods for image analysis procedures); color indicates nuclear cycle (see legend). Insets show log-linear plots of same data; slopes of fitted lines indicate a length constant of ∼0.15 EL from n.c. 8 onward. From n.c. 10 onward, GFP intensities for cortically localized dorsal nuclei only are shown for clarity. (C–D) Same data as in (A–B) but plotted as a function of developmental time (nuclear cycles) with copper shading indicating AP position as fraction of EL. (C) Nuclear GFP intensities as a function of time. Lines connect binned means of 50 equally spaced intervals along the AP axis. (D) Binned means normalized to the maximum attained in each bin during development, plotted as a function of time.

**Figure 8:**
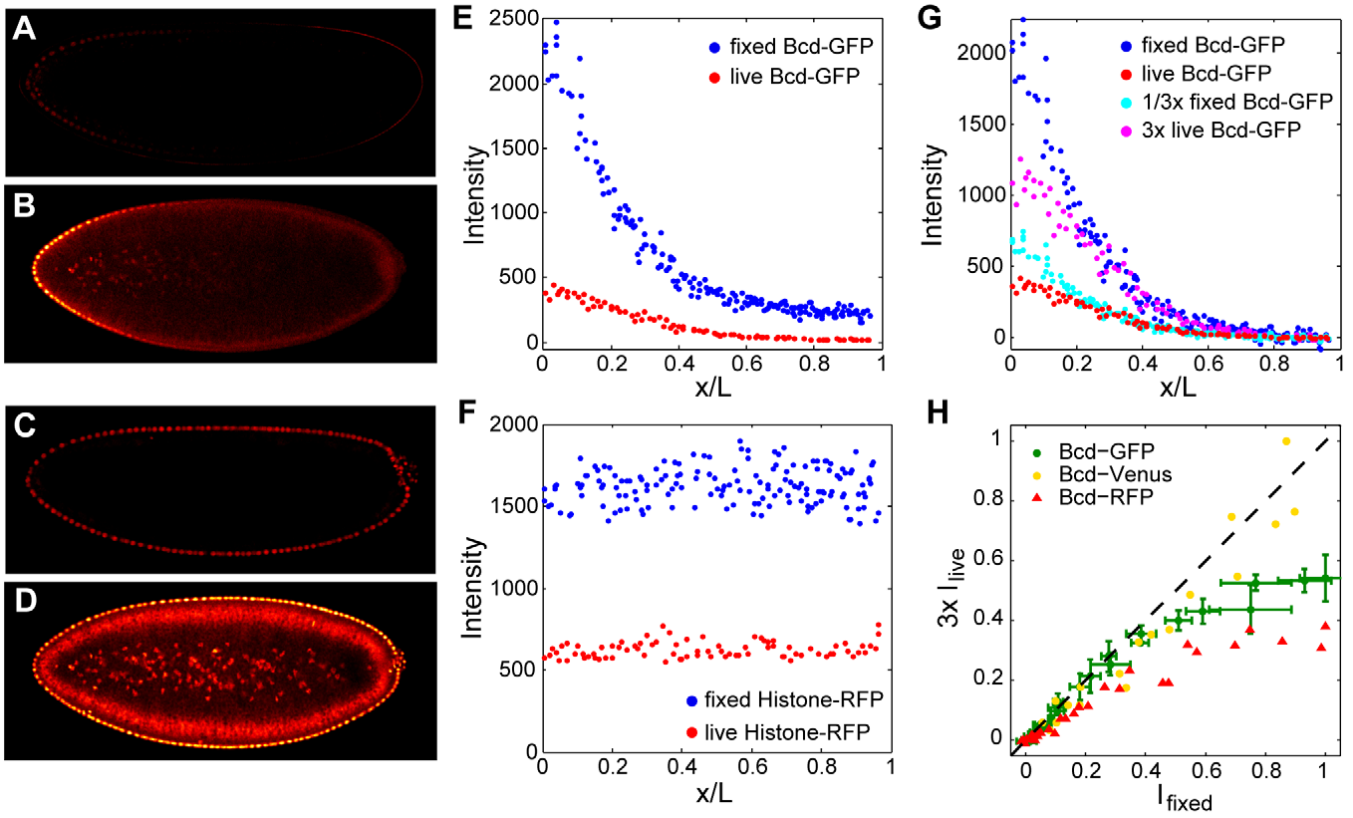
Bcd-GFP gradients in fixed and living embryos. Living Bcd-GFP (A–B) and Histone-RFP embryos (C–D) were imaged at blastoderm stages using confocal microscopy. Embryos were fixed within 3 min of live imaging, then subsequently re-imaged under the same microscopy settings (midsagittal slices of the same embryo at n.c. 13 are shown live in (A, C) and fixed in (B, D)). (E–F) Mean nuclear fluorescence intensities in living (red) and fixed (blue) embryos expressing Bcd-GFP (E) and Histone-RFP (F) as a function of position along AP axis. (G) Live and fixed Bcd-GFP gradients rescaled by 3-fold. (H) Scatter plot of fixed (*x*-axis) and 3-fold rescaled live (*y*-axis) mean intensities in equally spaced bins along the AP axis in embryos expressing Bcd fusions to GFP, Venus, or RFP. Dashed line indicates 1-to-1 correspondence. Error bars for Bcd-GFP are standard deviations within each bin. To plot curves on the same axes, values were normalized to maximum.

To quantify the Bcd gradient at these early stages, we extracted nuclear fluorescence intensities from embryos expressing both Bcd-GFP and Histone-RFP. The latter provides a nominally uniform fluorescence signal in all nuclei and serves as a reference to normalize Bcd-GFP values at different optical depths in the sample (see Materials and Methods). Corrected nuclear Bcd-GFP gradients between n.c. 6 and n.c. 11 show that at all positions along the AP axis, nuclear Bcd-GFP rises continuously (Figure 7A and 7C). The shape of the gradient, however, does not change (see slopes on log-linear inset to Figure 7A), whereas its amplitude continues to rise and increases by a factor of ∼3 over the course of n.c. 6–11. The embryo-wide increase in Bcd-GFP is abruptly halted along the entire AP axis at n.c. 11–12, by which time nuclei have attained Bcd concentrations >90% of their maximal values. Therefore, the nuclear gradient forms over approximately 50 min between n.c. 6 and n.c. 11. During n.c. 11 and 12 nuclear Bcd-GFP concentration remains within ∼95% of its maximum along the whole AP extent. After n.c. 12, nuclear concentrations drop, starting at the anterior; by n.c. 13, values in the anterior 20% of the embryo are approximately 80%–90% of their maxima at n.c. 12. At the beginning of n.c. 14, all positions along the AP axis decay to around 70%–85% of the maximum (Figure 8D). Despite this decrease in nuclear Bcd, the total amount of Bcd protein still rises through n.c. 13 (Figure S11) [9], consistent with continued protein production until mRNA degradation.

These observations of gradient dynamics differ sharply from the *bcd* mRNA distribution, which at no time resembles an exponentially decreasing gradient reaching to >75% EL, such as observed for Bcd-GFP from n.c. 9 onward. Bcd-GFP is visible in nuclei positioned at 50% EL and beyond from n.c. 8 onward, yet at these positions we observe essentially no mRNA particles. Instead, the protein gradient can only be accounted for if protein moves towards the posterior from anteriorly localized mRNA.

### Delayed GFP Maturation Can Account for Apparent Discrepancy Between Gradients in Fixed and Live Tissue and Provides Further Evidence for Cytoplasmic Protein Movement

By live imaging, nuclear Bcd-GFP levels appear relatively stable after the 10^th^ mitosis [9], in contrast to our observations in fixed tissue. Additionally, we observe that the decay length of fixed gradients is approximately 0.15 EL (Figure 8A inset), compared to a decay length of around 0.2 EL in live embryos [9]. These differences do not result from the use of different microscopy methods (Figure S12) and therefore must arise from the fixation procedure.

To determine the effect of fixation on Bcd-GFP fluorescence, we imaged living Bcd-GFP and Histone-RFP embryos at blastoderm stages, then fixed the embryos within 3 min of live imaging, and subsequently re-imaged under the same microscopy settings (Figure 8A–D). We found that fixation increases the overall brightness by about 3-fold (Figure 8E–F), possibly as a result of increased transparency of fixed material mounted in a glycerol-based medium. However, rescaling live or fixed Bcd-GFP gradients by a factor of 3 reveals qualitatively different gradient shapes in the anterior 25% of the egg, wherein fixed gradients exhibit an additional increase in relative intensity (Figure 8G). This remaining difference could be accounted for if newly synthesized Bcd-GFP is not immediately visible but becomes fluorescent after fixation. This phenomenon can be attributed to the well-known process of GFP maturation ([42] and refs therein). To test whether delayed maturation can affect the appearance of Bcd gradients detected by fluorescent protein fusions, we imaged transgenic embryos expressing protein fusions of Bcd with either the rapidly maturing GFP derivative Venus [43] or the more slowly maturing mRFP [44]. We found that after 3-fold rescaling, live and fixed Bcd-Venus gradients exhibited similar appearances, consistent with the rapid attainment of fluorescence by Venus (Figures 8H, S13A–C). In contrast, rescaled live Bcd-mRFP gradients showed poor correspondence with fixed gradients along the majority of the AP axis (Figures 8H, S13D–F). This result supports the view that delayed maturation can alter the appearance of the Bcd protein gradient. In addition, by incorporating delayed maturation into a simulation of gradient formation (see below), we could qualitatively recapture the stable nuclear gradients observed in live embryos at late blastoderm (Figure S14). We note that previous work has determined that gradients of equivalent shape are observed upon quantification of GFP fluorescence and anti-GFP immunostaining in the same embryo [45]. Moreover, the distribution of Bcd protein detected by anti-Bcd antibodies does not differ between wild-type and embryos in which Bcd-GFP is the only source of Bcd protein [8].

A maturation-based explanation supports the idea of protein movement away from anterior mRNA. With delayed maturation, immature fluorescent protein synthesized at the anterior would tend to attain fluorescence while en route toward the posterior. As a result, in a live embryo, the fraction of immature eGFP would be higher in the anterior than the posterior, and allowing additional time for eGFP to attain fluorescence would then disproportionately increase anterior fluorescence compared to posterior, as we observe by fixation.

### A Spatially Distributed Source Is Necessary to Account for Observed Protein Gradient Dynamics

Diffusion represents a simple plausible mechanism of protein transport away from the anterior mRNA source, and diffusion-based models such as the SDD model provide a straightforward mathematical framework for describing gradient establishment. Previous modeling efforts have treated the site of synthesis either as an anteriorly localized point source [9],[46]–[49] or as conjectured anterior domains [11],[50]–[53]. Having characterized the actual distribution of *bcd* RNA, we asked whether that distribution is a necessary component of gradient establishment by performing numerical reaction-diffusion simulations of gradient formation. We compared our measured nuclear gradients to protein distributions predicted to arise from either a realistic mRNA distribution or an anterior point source. We find that the combination of a non-monotonic time course for nuclear gradient amplitude (Figure 7D) and a relatively stable length constant between n.c. 8 and 14 (Figure 7B) precludes a classical SDD-type model in which the biophysical parameters of translation rate S, degradation rate τ, and diffusion D are kept constant at all times. Therefore, to reproduce the measured nuclear concentrations, our simulations utilized an “extended SDD” model which allowed some subset of these parameters to change in time (see Materials and Methods).

**Figure 9:**
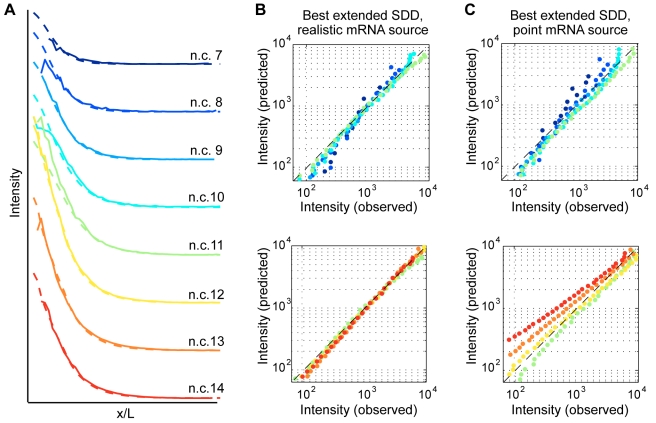
Extended SDD models with realistic 3d geometry and source distribution. (A) A well-fitting model with parameters as in Figure S10: solid lines are measured Bcd protein gradients from n.c. 7 (dark blue) to n.c. 14 (red); dashed line come from a single model fit. (B) Log-log plot of the measurement versus the model prediction at different developmental times (colors as in (A)); dashed black line corresponds to perfect overlap. (C) The best-fitting model when an mRNA point source at the anterior pole is assumed. Despite having the same freedom to pick the model parameters as in (B), the best-found model for the point source deviates from the data substantially.

Using the measured *bcd* mRNA distribution for the source, we found excellent fits between predicted and observed nuclear protein gradients from n.c. 7 to n.c. 14 (Figure 9A–B), as determined by a χ^2^-error measure (Figure S15). Conversely, if the model assumes a point-source geometry at the anterior of the embryo, an equivalent search through the parameter space of extended SDD models fails to adequately reproduce our observed Bcd protein data (Figure 9C). In particular, at early time points, the point source causes anterior protein levels to rise inappropriately high to attain a good overall fit. Additionally, the disperse source allows the model to better predict the late decrease in gradient amplitude. Indeed, estimates of total protein based on available measurements suggest that total Bcd decreases between n.c. 13 and n.c. 14 despite the doubling of nuclei (Figure S11). Simultaneously with the global decrease in protein levels, we see a degradation of disperse mRNA. Our simulation can account for the observed drop in protein distribution only if we use the realistic mRNA geometry during this decay period, rather than a point source (Figure 9B, C). These numeric results demonstrate that realistic mRNA distributions significantly outperform the point source geometry in reproducing the observed protein gradient dynamics.

## Discussion

Prior work demonstrating the precision in Bcd-GFP nuclear gradients [9] prompted us to examine two aspects underlying the formation of the Bcd gradient. First, we adapted a novel mRNA labeling methodology to the *Drosophila* embryo to examine global *bcd* mRNA dynamics [25]. Second, we quantified the formation of the nuclear Bcd-GFP gradient in fixed embryos during the entire period from fertilization to cellularization. A comparison of mRNA and protein gradient dynamics reveals that protein movement must occur away from the anteriorly localized source. Further, by analyzing numerical simulations of protein gradient establishment, we find that simple SDD models fail to account for the observed protein gradient dynamics. In contrast, extended SDD models utilizing the measured mRNA source distribution account for the data well, thus implicating a role for the disperse *bcd* mRNA distribution in gradient formation.

### A Sensitive FISH Method Captures Global *bcd* mRNA Particle Dynamics

Our method enabled us to quantify the global *bcd* mRNA particle distribution as a function of developmental time. The detection of dim particles even in the far posterior of the embryo demonstrates the improved sensitivity of our protocol. In addition, the high degree of spatial resolution allows us to document the characteristics of individual particles over developmental time. The method is sufficiently sensitive that we can successfully discriminate mRNA degradation at n.c. 14 from particle dissolution by the beginning of blastoderm stages.

Until n.c. 7, we see intensity distributions clearly separated from the noise peak (Figure S4), yielding robust estimates of particle counts with very low deviations (∼10%) across embryos (Figure 4C). As the particles disassemble after n.c. 10, the weakest particles (possibly single *bcd* mRNA molecules) move closer to the detection threshold, which could, in principle, lead to a significant number of missed particles. Therefore our counting results for late embryos should be strictly interpreted as lower bounds on the actual number of particles in late embryos. However, the fact that the total particle light is approximately conserved (Figure 4D), and that we can clearly identify an explicit mRNA degradation event during n.c. 14, strongly argue for the possibility that even in later embryos we are in fact recovering most of the particles and their cumulative intensity.

The biological mechanism(s) mediating mRNA particle movement during embryogenesis are as yet unknown. Previous work has shown that the reorganization of the cortical actin cytoskeleton upon egg activation allows the dispersion of *bcd* away from the oocyte cortex [30]. Whether endogenous *bcd* particles continue to localize with other cytoskeletal elements, such as microtubules, during embryogenesis is unknown, although in vitro synthesized mRNA containing the *bcd* 3′ UTR localization signal associates with microtubules when injected into embryos [32]. Redistribution of *bcd* RNP binding sites on microtubules might provide a method of scaling mRNA spatial distribution with different egg sizes. Such scaling could then potentially influence the observed scaling of the protein gradient [21].

It is interesting that *bcd* particles containing more mRNAs tend to be localized closer to the anterior than weakly fluorescent particles. Possibly, brighter *bcd* RNP complexes possess greater numbers of protein components required for establishment and/or maintenance of anterior localization, such as Staufen [5]. A greater content of Staufen (or other factors) would then lead to a greater probability of maintenance at the anterior. If so, then the slight posterior shift we observe may be linked to particle dissolution: as particles dissociate during development, they lose some fraction of localization components and tend to move posteriorly.

We observed weakly fluorescent particles in the posterior at all nuclear cycles. Such particles likely represent *bcd* RNA which failed to localize to the anterior during oogenesis, although we cannot formally rule out that such RNAs were in fact initially correctly localized and became posterior later. The fraction of *bcd* mRNA in the posterior 60% is near zero and thus contributes only negligibly to the protein gradient. Nevertheless, the embryo possesses a failsafe mechanism of suppressing *bcd* translation via Nanos activity in the posterior [36]. Such a failsafe might buffer the effects of environmental or genetic conditions that result in mislocalization of *bcd*.

### The Spatial Distribution of *bcd* mRNA Cannot Account for Protein Gradient Dynamics

Our observations of *bcd* mRNA are similar to the originally observed, steeply graded mRNA distributions of St. Johnston et al. [5]. In contrast, our results do not fully agree with a recent study which suggested that posteriorly directed *bcd* mRNA movement at the cortex accounts for the gradient of Bcd protein [19]. Our analysis is partially consistent with their findings: during early cleavages, *bcd* mRNA on the embryo surface does not extend far beyond ∼5% EL (Figure 2I–J), whereas surface particles are relatively dense even at 15% EL during syncytial stages (Figure 3A, 3C, 3G). However, the latter change is the result of bulk particle movement from the interior to the embryo surface. Such movement would not have been detected in the study by Spirov et al. [19], which did not succeed in labeling internally localized *bcd* mRNA in either early cleavage stage embryos or unfertilized eggs (Figures 2A–C, 6A–C of Spirov et al. 2009 [19]). This is reflected in the apparent increase in total mRNA fluorescence observed in that study between n.c. 3 and 10, a behavior impossible for strictly maternally supplied transcripts, but consistent with the arrival of previously undetected internal *bcd* mRNA at the embryo cortex. Since the amount of mRNA falls to nearly zero too close to the anterior to account for the presence of Bcd-GFP in nuclei at nearly 70% EL, we conclude that the spatial distributions of *bcd* mRNA and protein are at no point in time rescaled versions of each other. As we note above, Bcd-GFP is evident in nuclei positioned at 50% EL and beyond beginning at n.c. 8, yet at these positions, the mRNA concentration is essentially zero. Similarly, Bcd-GFP is retained in yolk nuclei after *bcd* mRNA has relocalized cortically (Figures 6E–I, S16). Furthermore, if we suppose that Bcd is unstable so that the local concentration of *bcd* mRNA dictates protein concentration (as proposed recently [19]), then the amount of mRNA at, e.g., 20% EL during n.c. 7 should be the same as the amount of mRNA at 50% EL at n.c. 11, given that nuclear Bcd-GFP concentrations are approximately the same in those locations at those times; however, this is not what we observe.

### Modeling Implicates *bcd* mRNA Distribution in Protein Gradient Dynamics

Our observations present a revised description of protein gradient dynamics in which along the entire AP axis, nuclear Bcd concentrations reach their maximum values by n.c. 11. Previous work cited the difficulties of a simple SDD-based model of gradient formation given the apparent slow diffusion coefficient D of Bcd-GFP (∼0.3 µm^2^/s) as measured in the blastoderm cytoplasm by photobleaching [9]. More recent measurements by fluorescence correlation spectroscopy have estimated D≈7 µm^2^/s, which in principle would be rapid enough to establish a stable gradient prior to n.c. 9 [22],[23]. The observations presented here, however, indicate that nuclear Bcd-GFP continues evolving beyond that point in a non-monotonic manner. Taken together with recent work suggesting that nuclei function as passive absorbers of local Bcd [40], these results would appear to preclude a classical SDD model with constant parameters in which the Bcd distribution reaches a stable state.

Simple extensions of SDD provide the mathematical framework within which to model protein movements from disperse sources. While such minimally extended models do not explicitly include the role of nuclei and non-diffusive modes of protein transport that might play an important role [51], they nevertheless allowed us to discover parameter sets that successfully predict the observed protein gradient when incorporating the observed, disperse mRNA sources; in contrast, the best fit parameters utilizing only an anterior point source do not match data as well. This result can be understood by noting that while the protein gradient beyond the AP extent of its mRNA source is not influenced much by the geometry of the source, the same does not hold for the anterior part of protein gradient that overlaps with mRNA.

We note that our measurements are limited to nuclei and that we do not explicitly account for cytoplasmic Bcd-GFP. Using currently available estimates [9], it is likely that total nuclear and cytoplasmic Bcd-GFP continue rising through n.c. 13 (Figure S11). We also note that n.c. 13 and 14 exhibit extended interphases, during which nuclear diameter increases and Bcd-GFP concentrations fall as a result [9]. These changes in Bcd levels both during and between interphases pose a challenge to understanding the transcriptional readout of Bcd concentrations. The well-documented dependence of Bcd target gene expression on Bcd levels implies that the readout process must be insensitive to such global changes in nuclear Bcd concentration. The nature of the underlying readout process, and how it achieves the observed reproducibility and accuracy of transcriptional regulation, will be the subject of future experiments.

## Materials and Methods

### FISH of *Drosophila* Embryos

OregonR embryos were fixed as described [26] in 10% paraformaldehyde and stored in methanol. We performed fluorescent in situ hybridization as described [25], using AlexaFluor 514-conjugated probes complementary to the *bcd* open reading frame diluted in a solution of 35% formamide, 2× SCC, 0.1% Tween-20. Embryos were mounted in Aqua-Poly/Mount (Polysciences). For high resolution imaging, we used a 100× HCX PL APO CS NA 1.46 oil immersion objective on a Leica SP5 laser-scanning confocal microscope with 514 nm excitation wavelength at 2048×2048 pixels (linear dimension of 75.7 nm) and z-spacing of 420 nm, and obtained one anterior and one posterior stack of each embryo. Low resolution images of the midsagittal plane were taken with a 20× HC PL APO NA 0.7 oil immersion objective at 1024×500 pixels.

### FISH Image Analysis

To correct for drift of the microscope stage in the xy-plane, we first realigned the z-slices by starting at the midsaggital plane (z = 0) and shifted successive slices such that the pixel-wise cross-correlation in raw intensity between slice z and z+1 was maximized. To normalize for changes in optical medium with depth z, we selected 10^5^ small image patches from each posterior z-slice, computed the mean and variance in raw intensity from each patch, and fit a linear relation between the two; if noise in the image were purely uncorrelated Poisson shot noise, the slope would yield a conversion factor between the raw microscope signal and the photon counts. We then normalized every z-slice by this factor, which changed slowly and systematically with z. Neither realignment nor normalization crucially affect our reported results.

We next filtered each z-slice by a balanced circular difference-of-Gaussians filter with center radius size of 1.5 pixels and surround size 2.5 pixels, roughly matched to the size of single *bcd* mRNA particle, and found the local maxima in the filtered images that exceed the detection threshold. These are *candidate particles*. If the detection threshold is too low, the number of candidate particles in the posterior stack rapidly increases due to false detections of noise and autofluorescence, as shown in Figure S4 (red line). We pick for the threshold a value just above this rapid increase and find that after normalization, a single threshold value can be chosen for all embryos.

Each candidate particle is then processed in turn: starting with the brightest unprocessed candidate C in the z-stack, successive nearby z-slices are examined for other candidates that appear at the same x-y location due to the axially extended PSF of the microscope (Figure S3); these candidates are considered as shadows of C, not independent particles. By identifying candidates and their shadows on successive planes, we find that the average light intensity profile of the mRNA particle in the axial (z) direction is approximately Gaussian, with decay length of about 1 slice. Given our detection and microscopy settings, we therefore expect to observe 1–2 shadows for each true mRNA particle. Next, we proceed by fitting the light intensity of each candidate particle as a 2D elliptical Gaussian profile plus constant background (Figure S2). In detail, we clip 9×9 pixel patches centered on the candidate and fit a light intensity model I(x,y) = offset+amplitude * Gaussian(x−x_c_, y−y_c_, r_x_, r_y_, theta), where x_c_ and y_c_ are the light intensity center coordinates, r_x_ and r_y_ are orthogonal ellipse axes, with r_x_ inclined by angle theta to the horizontal. If within the 9×9 patch other candidates are identified, we clip out a larger patch and simultaneously fit all nearby candidates to properly resolve their radii and amplitudes (Figure S2). Each candidate particle in the list has thus been concisely described by parameters (x_c_, y_c_, r_x_, r_y_, offset, amplitude, number of shadows).

Since the threshold is set as low as possible to capture all mRNA signal, the candidates might include false positive detections. To eliminate these, we require that (i) r_x_, r_y_ be above 0.7 and below 2.5 pixels, (ii) the amplitude to exceed the background offset, and (iii) the number of shadows be at least 1. Detection threshold and number of shadows are the most stringent criteria (Figure S4, blue and cyan lines), while (i), (ii) are far less restrictive. Candidates passing these criteria are considered as mRNA particles, which exhibit stereotyped, circular shape (Figure 3A–B); their intensity is proportional to amplitude * r_x_ * r_y_. We find a very good linear correspondence between the intensity and difference-of-Gaussians filter value for each particle, and the results are robust to the choice of either of these intensity measures as the thresholded variable. Figures 3C and 4C–D report the statistics of particle counts and intensities (distributions are normalized to unit area).

To extract the spatial distributions, we must translate the x_c_, y_c_ location of each particle detected at 100× into the projection along AP axis. Using image manipulation, we register the 20× and 100× images of midsaggital plane and extract automatically the coordinates of the AP axis, of length L, from 20× images. This allows us to compute for each particle its x/L relative coordinate, and to construct distributions in Figure 3D–F. In 4A we similarly compute the density of mRNA particles per unit area in midsaggital plane in thin slices parallel to embryo surface at depth d/L.

### Protein Gradient Quantification

Embryos from *yw egfp-bcd*; *His2Av-mRFP1*; *bcd^E1^* females (obtained by crossing [9],[54]) were fixed in 6.7% paraformaldehyde and the vitelline membrane removed by hand. DAPI-stained embryos were mounted in Aqua-Poly/Mount and imaged using a 20× HC PL APO Gly 0.7 NA objective on a Leica SP5 laser-scanning microscope with excitation wavelengths of 405 nm (DAPI), 488 nm (eGFP), and 561 nm (mRFP). Image stacks were obtained at 2048×2048 pixels (linear dimension 445 nm) with z interval of 3.5 µm spanning approximately 75% of embryo z-thickness. Custom software employed smoothened DAPI and RFP images to identify nuclei by thresholding in individual z slices, as well as embryo edges [11], resulting in 3d nuclear masks. For embryos at blastoderm stages, a watershed was applied to separate closely spaced objects. 3d masks were checked in parallel with GFP, RFP, and DAPI images to ensure detected objects consisted only of nuclei; inappropriate objects were discarded and each independent set of connected pixels was designated a nucleus. The mean GFP and RFP pixel intensities and centroid position for each nucleus were calculated. Histone-GFP and Histone-RFP mean nuclear intensities attenuate at similar rate as function of depth beneath the embryo surface. Assuming that all nuclei contain the same DNA content and therefore the same concentration of Histone-RFP, we used the attenuation in mean RFP intensity to apply a corrective factor to nuclei (as performed previously using anti-Histone antibodies [55]). We calculated autofluorescent background by comparing to OregonR embryos, observing that nuclei at 85%–95% EL in Bcd-GFP expressing embryos contain the same mean GFP intensity as fixed OregonR embryos imaged under the same conditions. In all plots except Figure 8E–F, we show attenuation corrected, background subtracted mean nuclear Bcd-GFP values.

For the experiment in Figure 8 and Figure S13, after live imaging, embryos were immediately transferred to fixative, processed, and imaged as described above. Embryos were handled individually to ensure we could match live and fixed images to the same embryo. Live and fixed images were taken under identical conditions.

### Simulations of Bcd Gradient Establishment

We performed numerical reaction-diffusion simulations of the extended SDD model on a 3d 10 µm grid. We adopted the embryo geometry from the BDTNP map [56], which represented our solution domain with no-flux boundary conditions. In the interior, we solved
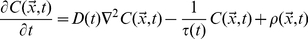
(1)where 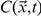 is the concentration of Bcd protein, and D, τ, and ρ are (nominally time-dependent) diffusion, degradation, and synthesis rates. The source ρ was taken to be a collection of measured *bcd* mRNA particle intensities; the positions of the particles were readjusted to undo the axial compression caused by the imaging protocol. We kept the source geometry unchanged for nuclear cycles for which we do not have explicit measurements. The total source strength was kept constant according to the observed constant total intensity with developmental time (Figure 4D). We extracted the temporal snapshots after time increments of 8 min each for n.c. 1 to 9, 9 min for n.c. 10, 10 min for n.c. 11, 12 min for n.c. 12, and 20 min for n.c. 13 and 14. The obtained concentration profiles were projected along the AP axis. As the units of measurement are arbitrary, the simulation gradients were rescaled with a single scale parameter to match as closely as possible the measurements for n.c. 7–12; the goodness of fit was defined as
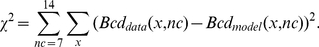
(2)Thus, models that have small 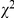 match the data better. We then asked how closely we can reproduce the observed gradients (without any assumption about the gradient equilibration and without any reference to previously measured D and τ parameters) within the classic SDD model, where D, τ are constant and ρ is given by the measurement, assuming continuous translation from the source. As expected from the non-monotonic temporal dependence of the gradient amplitude reported in Figure 7D, no combination of parameters for the classic SDD model fits the data well.

The most straightforward extension of the SDD model is to allow some subset of translation rate, degradation rate τ, and diffusion constant D parameters to vary in time. To guide our exploration of such extended SDD models, we first noted that protein gradients at different times are rescaled versions of each other, i.e. the length scale is approximately constant from n.c. 8 onwards (relative change is less than 10% from n.c. 8 till n.c. 14), while the gradient amplitude increases by ∼3-fold from n.c. 8 to the time it reaches its peak in n.c. 12, and decreases to about 3/4 of the value in n.c. 14. Simultaneously satisfying both constraints, i.e. preserving the decay length and properly scaling the gradient amplitude as a function of time, is challenging in the framework of such extended SDD models. Decrease in gradient amplitude at later times can be generated in models in which the source turns off at n.c. 12, but if diffusion still remains active, the gradients are very flat at low x/L, inconsistent with the data. Models in which the decrease in gradient amplitude after n.c. 12 is generated by turning on degradation later yield gradients that match the data reasonably at low x/L, but do not preserve the exponential profile with a conserved decay constant across developmental time; even models in which degradation is present during the whole developmental time, but can accelerate to a higher value in blastoderm stages, do not fit the data well. Furthermore, to account for low gradient amplitudes at n.c. 7 and 8, the source needs to be turned on late, i.e. for most of the models around n.c. 5 or 6.

With these observations in mind we carried out an exhaustive search for D, τ, and the start and end of *bcd* mRNA translation, to find the set of parameters minimizing 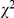 error (Figure S15); the source term is set to 0 before the start and after the end of translation. A well-fitting model could only be found if additionally the diffusion constant was allowed to change during developmental time. The best match to our data is obtained for models where (i) the source is active only during n.c. 6–12, (ii) degradation is essentially constant with τ∼100–140 min, and, (iii) the diffusion constant (initially set to D∼3 µm^2^/s) decreases sharply to 0 at n.c. 11–12 (Figure 9C–D) (a decrease to a small value, e.g. 0.3 µm^2^/s, instead of 0 yields an even better fit). The 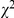 of this model is approximately one half of the classic SDD with realistic source.

To assess the effects of the source geometry on the protein gradients, we replaced our measured source by a point source localized at the anterior pole, and repeated the parameter search by varying D, τ, and the start and stop times for translation. The best fit in this scenario deviates substantially from the measured gradients (Figure 9D), mostly because simulations tend to overestimate the steepness of the gradient close to x/L = 0. The 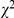 of this model is 2 times larger than the best extended SDD model with a realistic source.

To assess the effects of GFP maturation (Figure S14), 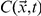 in equation (1) represented immature Bcd-GFP and we used an additional reaction-diffusion equation for the mature species without the source (ρ) term, but including a conversion term 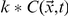 from the immature to mature species with rate *k*.

We emphasize that we employ these simulations for the express purpose of exploring the potential of disparate mRNA geometries to impact protein gradient formation. Our simulations represent coarse-grained descriptions of all processes that might impact protein production, stability, and diffusion. Multiple events could influence protein movement, such as advective transport, nuclear import/export dynamics, and/or changes in numbers or affinities of Bcd binding sites [9],[48],[51],[53],[57],[58]. As such, our model does not distinguish between these, and thus it does not explicitly support the notion that Bcd movement occurs only via thermally driven random molecular motion.

Likewise, within our model, protein production and stability are bound by two assumptions: first, that translation occurs from particles in proportion to their measured fluorescence intensities; and second, that nuclear Bcd-GFP gradients at all times reflect rescaled versions of the underlying cytoplasmic gradient [9],[40]. Because we observe nuclear accumulation of Bcd-GFP beginning at n.c. 6, it is unsurprising that we obtained better fits in simulations where protein production does not begin immediately upon fertilization (Figure S15). However, absent additional data between n.c. 1 and 5, we cannot advocate that translation of *bcd* mRNA necessarily begins at a later time point, although this would be consistent with previous proposals [41].

Similarly, at late blastoderm, simulating an apparent gradient decay while preserving the gradient shape requires that both production and effective diffusion decrease (see Materials and Methods, Figure S15). However, we do not conclude that either production or diffusion actually slow over time, as our model does not explicitly incorporate any mechanisms that could in principle affect the distribution of protein among nuclei (e.g., the geometric increase in the number of nuclei, Bcd trapping within local nuclear neighborhoods [51],[53], or the possibility that the nuclear-cytoplasmic ratio of Bcd in an equilibrated nucleus could change over time).

## Supporting Information

Figure S1 Low magnification *bcd* FISH with multiple oligonucleotides at n.c. 6–14. Low magnification midsagittal slices of embryos hybridized with fluorescently labeled *bcd* oligonucleotides at n.c. 4 (A), interphase 6 (B), mitosis 6 (C), n.c. 7 (D), n.c. 11 (E), and n.c. 13 (F) and n.c. 14 (early, G, and late, H). Green, *bcd* mRNA; blue, DAPI. Scale bar: 50 µm.
(2.22 MB TIF)

Figure S2 Example particle detection with custom analysis software. (A) A single well isolated particle detected as a local maximum of light intensity. Crosshairs indicate major and minor axes. (A′) Fitted elliptical Gaussian intensity distribution of the particle detected in (A). (B) Two overlapping spots within the red square are distinguished by software and represented by fitted Gaussians in (B′). (C) z-slices from 0.4 µm above (top) to 1.2 µm below (bottom) the image in panel (B) (red boxed panel). The position of the particle denoted by crosshairs in (B) is marked by boxed red dot. Cyan dots indicate all detected particles. The particle shown in (B) and its shadows on neighboring z-planes are indicated by the blue circled dot.
(0.18 MB TIF)

Figure S3 Point spread function. (A) Sequential z-slice confocal images of diffraction-limited, 200 **µ**m diameter fluorescent beads suspended in acrylamide. (B) Particle diameter (in pixels) of 345 beads measured by detection algorithm.
(0.13 MB TIF)

Figure S4 Particle detection criteria. Each panel shows a histogram of the balanced difference-of-Gaussians (DoG) filter values used to detect candidate particles (see Materials and Methods) from n.c. 3 to late n.c. 14. High values on *x*-axis correspond to particle candidates at very high contrast and thus high signal-to-noise ratio; low values correspond to dim particle candidates barely different from the background. Each line denotes analysis of one embryo. Red lines: the histograms for embryo posteriors. Essentially no candidates are detected for which the filter value exceeds ∼150 (this was used as the detection threshold for all embryos in our method; note the log scale on the *x*-axis). For smaller values of the threshold the number of candidates would explode as the detection algorithm starts picking up noise (note the sharp rise for small filter values). Blue lines: the histograms for embryo anteriors. For low filter values, the same explosion in noise detections is observed as in the posteriors. However, there is a clear excess of signal at high filter values due to true mRNA particle detections (the peak in the histograms above ∼150). Cyan lines: the histogram of filter values for embryo anteriors for only those particle candidates that are detected on at least 2 *z*-slices. This is one of the additional criteria used in our particle detection procedure (see Materials and Methods) that clearly excludes the explosive growth in false positives for low filter values but retains all detections at high filter values (note the overlap of blue and cyan curves for high filter values).
(0.28 MB TIF)

Figure S5 FISH background fluorescence. (A–C) Wild-type (Oregon-R) embryo at n.c. 5 processed for FISH without probes. (D–F) Embryo at the second mitosis processed for FISH with probes against the purely zygotically expressed gene *giant*. Scale bars: (A) 50 µm, (B) 25 µm, (C) 2 µm.
(4.03 MB TIF)

Figure S6 *bcd* mRNA particle number as a function of position along the AP axis. Shown are probability distributions of particle counts (A) and the cumulative distribution of particles (B) as a function of fractional distance along AP axis and nuclear cycle (color). The dataset and color code are equivalent to that shown in Figure 3.
(0.23 MB TIF)

Figure S7 *bcd* mRNA particle distribution at n.c. 13. (A–F) n.c. 13 embryo near the midsagittal plane at the anterior (A) and posterior (F). Shaded boxes indicate magnified views (B–E) corresponding to cortex (upper) or core (lower). (F inset) Boxes indicate regions shown in (A) and (F). (G–L) Same embryo as (A–F), imaged at the nuclear layer. (H–K) Selected *z*-slices of boxed regions indicated in (G) and (L) showing apical (upper panels) and basal (lower panels) planes surrounding the nuclear layer (middle panels). Arrowheads indicate selected faint particles. Scale bars: 25 µm (A), 2 µm (B).
(2.47 MB TIF)

Figure S8 Low magnification FISH with multiple oligonucleotides in n.c. 3–6. Low magnification confocal images of midsagittal (A–C, F) or coronal (D–E) sections. Green, *bcd* mRNA; blue, DAPI. Scale bar: 50 µm.
(1.49 MB TIF)

Figure S9 Schematic representation of *bcd* mRNA particle 3d distribution at early and late nuclear cycles. Locations of identified mRNA particles from early (until n.c. 6, A–D) and late (after n.c. 10, E–H) embryos were used to construct average 3d particle density profiles and typical cross-sections. (A–D) Sections of the transverse (blue frame, A), coronal (yellow frame, B), and midsagittal (green frame, C) planes of the 3d distribution (D) of early embryos show a wedge-shaped distribution of mRNA with a high concentration at the anterior pole *A*; redder color indicates higher particle concentrations. (E–H) In late embryos, *bcd* mRNA distribution resembles a cup covering the anterior pole, extending slightly farther into the posterior. Note the high concentration of particles in the cortex evident from the sections (E–G).
(0.50 MB TIF)

Figure S10 Nuclear accumulation of Bcd-GFP is not evident at interphase 5. Shown are maximum z-projections to display nuclei in multiple focal planes. (A) Bcd-GFP, (B) DAPI. Boxed regions in (A) and (B) are shown in (C) and (E), respectively. (D) Outlines of nuclear DAPI staining superimposed onto the image in (C). Scale bars: 50 µm (A), 20 µm (C).
(0.88 MB TIF)

Figure S11 Estimated total Bcd during blastoderm stages. Nuclear volume (red curve) was calculated from previous measurements of nuclear diameter [9]. These values were multiplied by nuclear gradient amplitudes measured in this study (blue curve; error bars indicate measurement error of 15% observed in the anterior 50%), which were then divided by the fraction of Bcd localized to nuclei [9] to obtain total protein (green curve; error bars determined by applying 20% accuracy of nuclear Bcd estimates [9]).
(0.10 MB TIF)

Figure S12 Confocal and two photon microscopy yield similar Bcd-GFP gradients in fixed embryos. (A) Example image obtained using custom built two-photon microscope as described [9] on the surface of Bcd-GFP autofluorescence in a fixed embryo at n.c. 13. (B) The same embryo as in (A), imaged by single photon confocal microscopy. (C) Nuclear gradients were extracted from each image. Raw intensity confocal values are shown. For comparison, values obtained by two photon microscopy were rescaled.
(0.45 MB TIF)

Figure S13 Bcd-Venus and Bcd-mRFP live and fixed gradients. Bcd-Venus [40] (A, B) and Bcd-RFP [40] (D, E) embryos were imaged live (A, D), fixed, re-imaged (B, E), and gradients extracted (C, F) as in Figure 9. In a live embryo, the vitelline membrane exhibits autofluoresence in the red channel and outlines the embryo (D); nuclei are observed within this outline. The membrane is removed prior to fixed imaging (E).
(0.83 MB TIF)

Figure S14 Simulated GFP maturation. The simulation of Figure 9B modified such that Bcd-GFP is not visible until 20 min after translation (see Materials and Methods). Other parameters are the same as in Figures 9 and S15.
(0.10 MB TIF)

Figure S15 Exploration of parameter space for extended SDD modeling. Shown in grayscale are χ^2^–errors of fits of the model to measured mean Bcd protein gradients for n.c. 7–14; darker values correspond to smaller χ^2^ and better fits. (A) The starting and stopping times of *bcd* mRNA translation, T_Src_^off^ and T_Src_^on^ (*x*- and *y*-axes, respectively, in nuclear cycles), are varied for 4 scenarios, where diffusion is either always on or drops to zero in n.c. 11, 12, or 13 as denoted in each quadrant of (A). For each choice of these three timing parameters, we look for the best values for parameters D and τ. (B) D and τ search for T_Diff_^off^ = 12, T_Src_^on^ = 7, T_Src_^off^ = 13. The best fits are obtained with D = 3.1 µm^2^/s and τ = 100 min; these values are used in Figure 9.
(0.16 MB TIF)

Figure S16 Bcd-GFP levels in yolk nuclei at blastoderm stages. Individual data points indicate mean attenuation-corrected intensities for individual nuclei, compared to binned means of dorsal cortical nuclei at n.c. 11 (black line).
(0.15 MB TIF)

Movie S1 Confocal stack of an embryo at n.c. 4 labeled with *bcd* probes. Each image represents one z-slice with interval of 0.42 µm; total *z* thickness 5 µm. One pixel represents 75×75 nm.
(10.07 MB AVI)

Movie S2 Confocal stack of an embryo at n.c. 13. Each image represents one *z*-slice with interval of 0.42 µm; total *z* thickness 5 µm. One pixel represents 75 × 75 nm.
(10.07 MB AVI)

Table S1 Sequences of *bcd* antisense oligonucleotides.
(0.02 MB XLS)

## Abbreviations

3d: three dimensional
AED: after egg deposition
AP axis: anterior-posterior axis
Bcd: Bicoid
EL: egg length
FISH: fluorescent in situ hybridization
GFP: green fluorescent protein
mRFP: monomeric red fluorescent protein
mRNA: messenger ribonucleic acid
n.c.: nuclear mitotic cycle
PSF: point spread function
RNP: ribonucleoprotein
SDD: synthesis-diffusion-degradation
