# Supplementary figures and images for: The Formation of the Bicoid Morphogen Gradient Requires Protein Movement from Anteriorly Localized mRNA
Source: PLoS Biol. 2011 Mar 1;9(3):e1000596. doi: 10.1371/journal.pbio.1000596 (PMC3046954; doi:10.1371/journal.pbio.1000596)

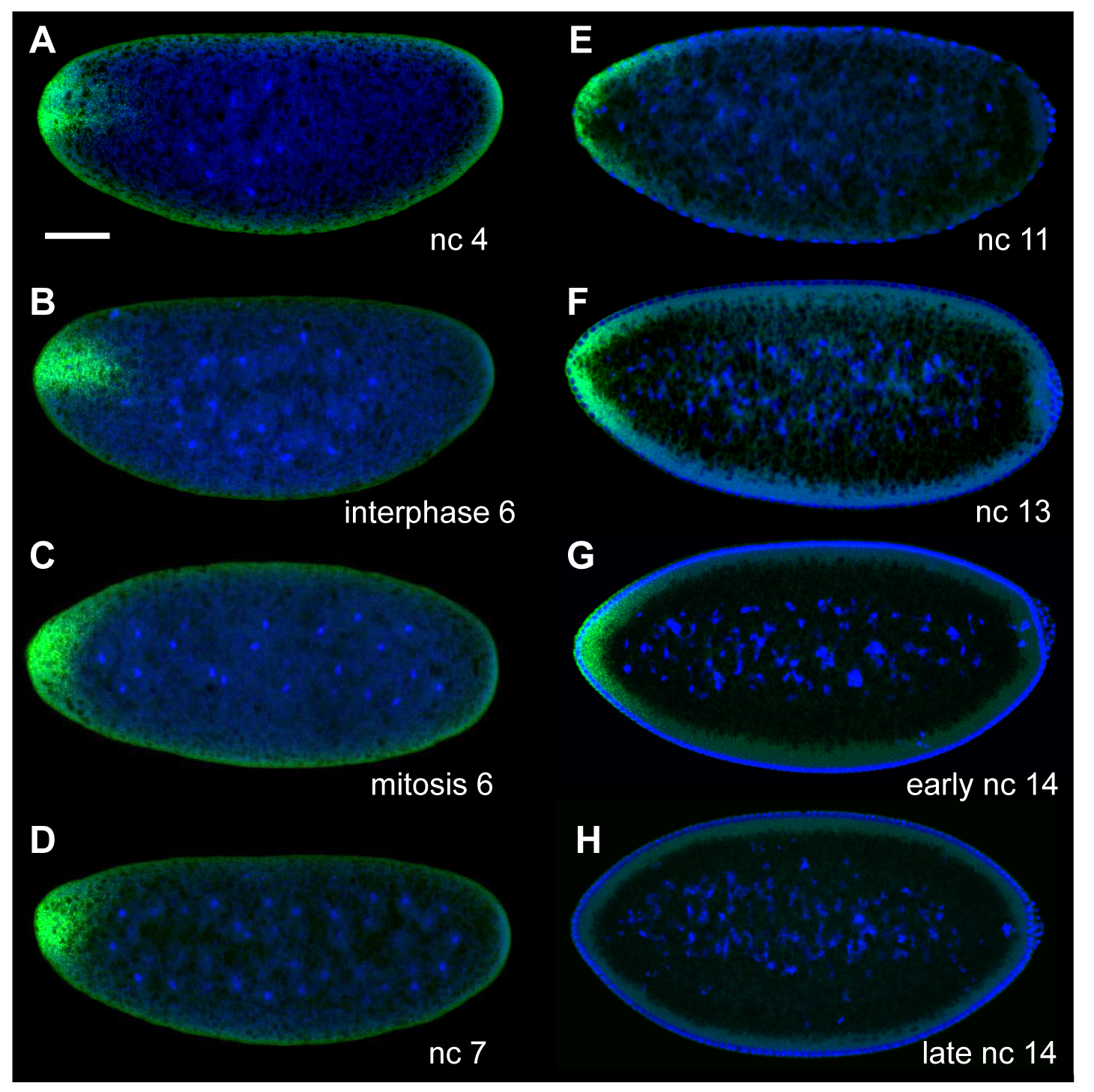

Supplement: Figure S1 — Low magnification bcd FISH with multiple oligonucleotides at n.c. 6–14. Low magnification midsagittal slices of embryos hybridized with fluorescently labeled bcd oligonucleotides at n.c. 4 (A), interphase 6 (B), mitosis 6 (C), n.c. 7 (D), n.c. 11 (E), and n.c. 13 (F) and n.c. 14 (early, G, and late, H). Green, bcd mRNA; blue, DAPI. Scale bar: 50 µm. (2.22 MB TIF) [file pbio.1000596.s001.tif]

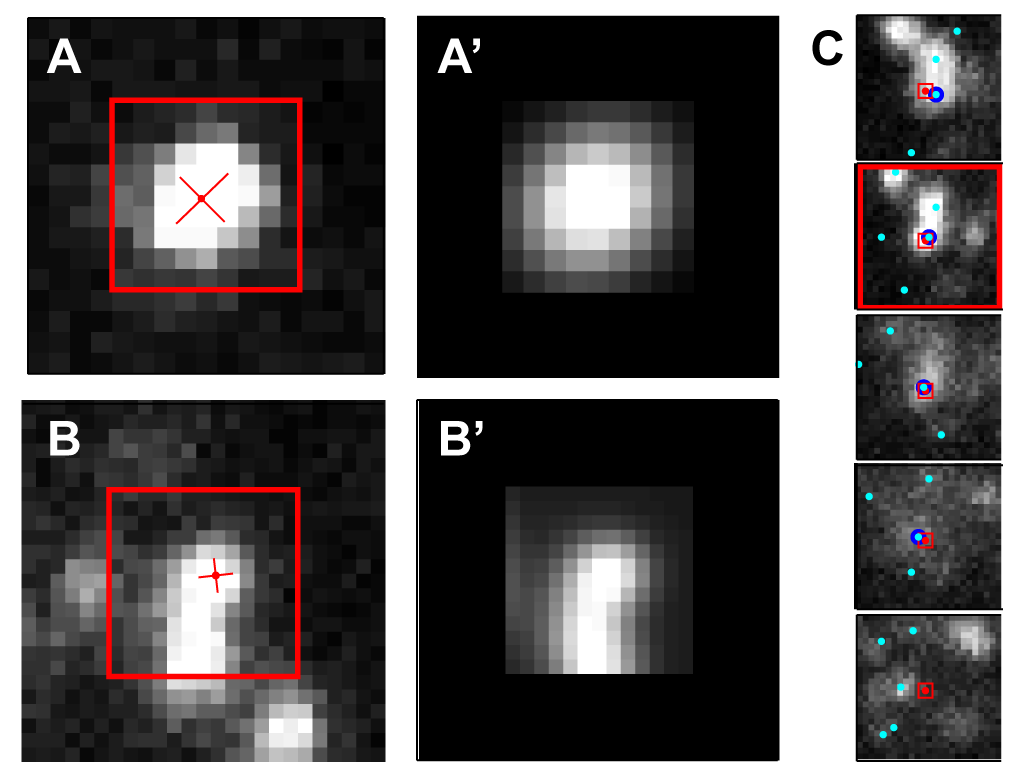

Supplement: Figure S2 — Example particle detection with custom analysis software. (A) A single well isolated particle detected as a local maximum of light intensity. Crosshairs indicate major and minor axes. (A′) Fitted elliptical Gaussian intensity distribution of the particle detected in (A). (B) Two overlapping spots within the red square are distinguished by software and represented by fitted Gaussians in (B′). (C) z-slices from 0.4 µm above (top) to 1.2 µm below (bottom) the image in panel (B) (red boxed panel). The position of the particle denoted by crosshairs in (B) is marked by boxed red dot. Cyan dots indicate all detected particles. The particle shown in (B) and its shadows on neighboring z-planes are indicated by the blue circled dot. (0.18 MB TIF) [file pbio.1000596.s002.tif]

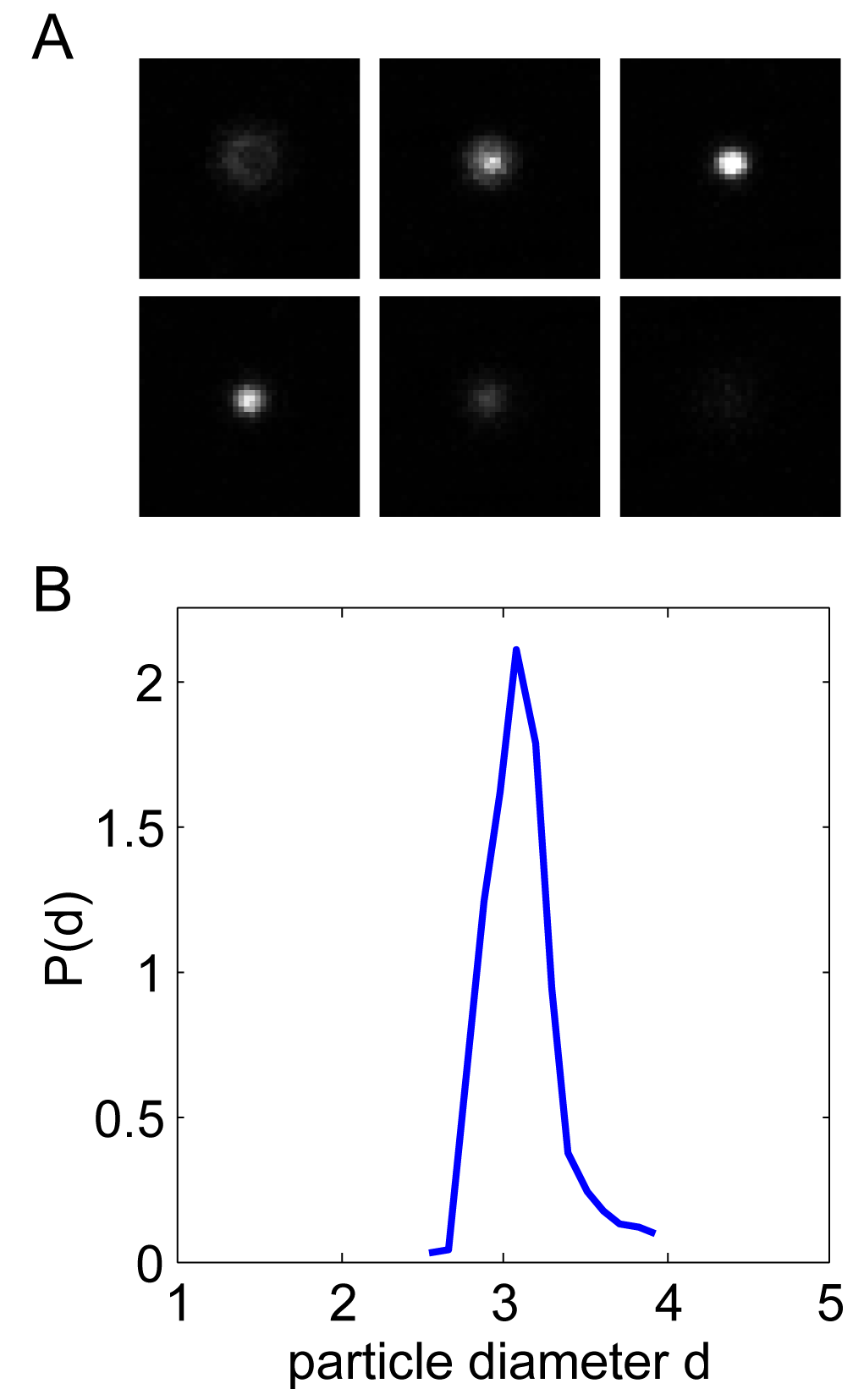

Supplement: Figure S3 — Point spread function. (A) Sequential z-slice confocal images of diffraction-limited, 200 µm diameter fluorescent beads suspended in acrylamide. (B) Particle diameter (in pixels) of 345 beads measured by detection algorithm. (0.13 MB TIF) [file pbio.1000596.s003.tif]

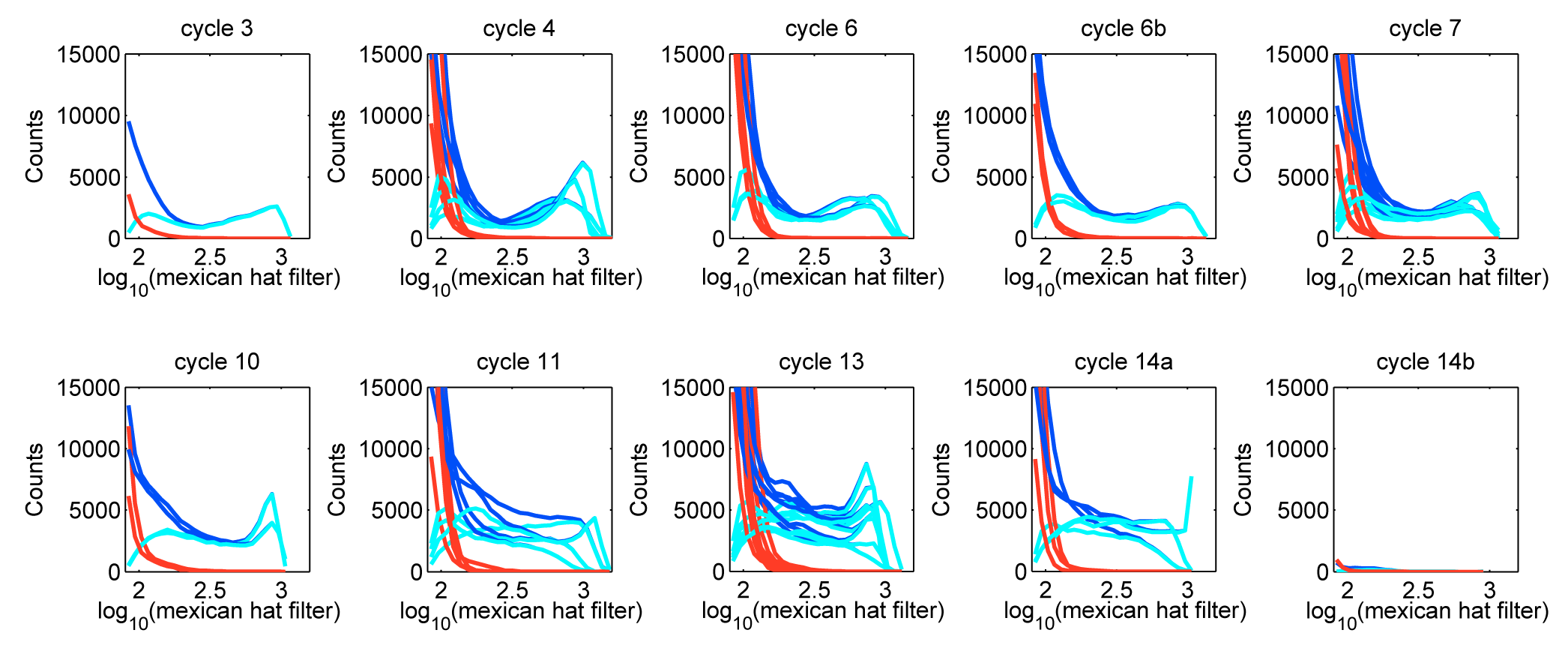

Supplement: Figure S4 — Particle detection criteria. Each panel shows a histogram of the balanced difference-of-Gaussians (DoG) filter values used to detect candidate particles (see Materials and Methods) from n.c. 3 to late n.c. 14. High values on x-axis correspond to particle candidates at very high contrast and thus high signal-to-noise ratio; low values correspond to dim particle candidates barely different from the background. Each line denotes analysis of one embryo. Red lines: the histograms for embryo posteriors. Essentially no candidates are detected for which the filter value exceeds ∼150 (this was used as the detection threshold for all embryos in our method; note the log scale on the x-axis). For smaller values of the threshold the number of candidates would explode as the detection algorithm starts picking up noise (note the sharp rise for small filter values). Blue lines: the histograms for embryo anteriors. For low filter values, the same explosion in noise detections is observed as in the posteriors. However, there is a clear excess of signal at high filter values due to true mRNA particle detections (the peak in the histograms above ∼150). Cyan lines: the histogram of filter values for embryo anteriors for only those particle candidates that are detected on at least 2 z-slices. This is one of the additional criteria used in our particle detection procedure (see Materials and Methods) that clearly excludes the explosive growth in false positives for low filter values but retains all detections at high filter values (note the overlap of blue and cyan curves for high filter values). (0.28 MB TIF) [file pbio.1000596.s004.tif]

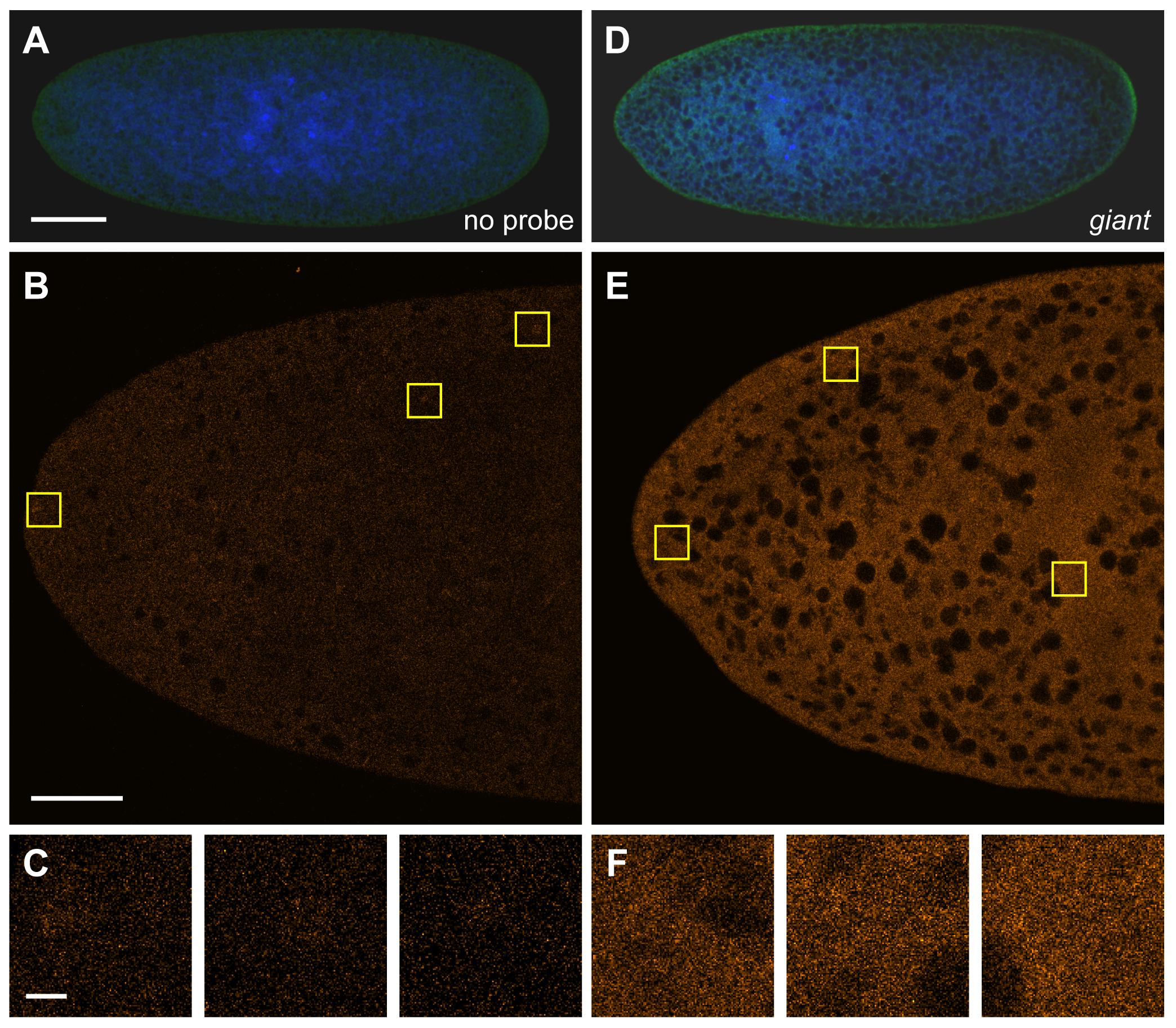

Supplement: Figure S5 — FISH background fluorescence. (A–C) Wild-type (Oregon-R) embryo at n.c. 5 processed for FISH without probes. (D–F) Embryo at the second mitosis processed for FISH with probes against the purely zygotically expressed gene giant. Scale bars: (A) 50 µm, (B) 25 µm, (C) 2 µm. (4.03 MB TIF) [file pbio.1000596.s005.tif]

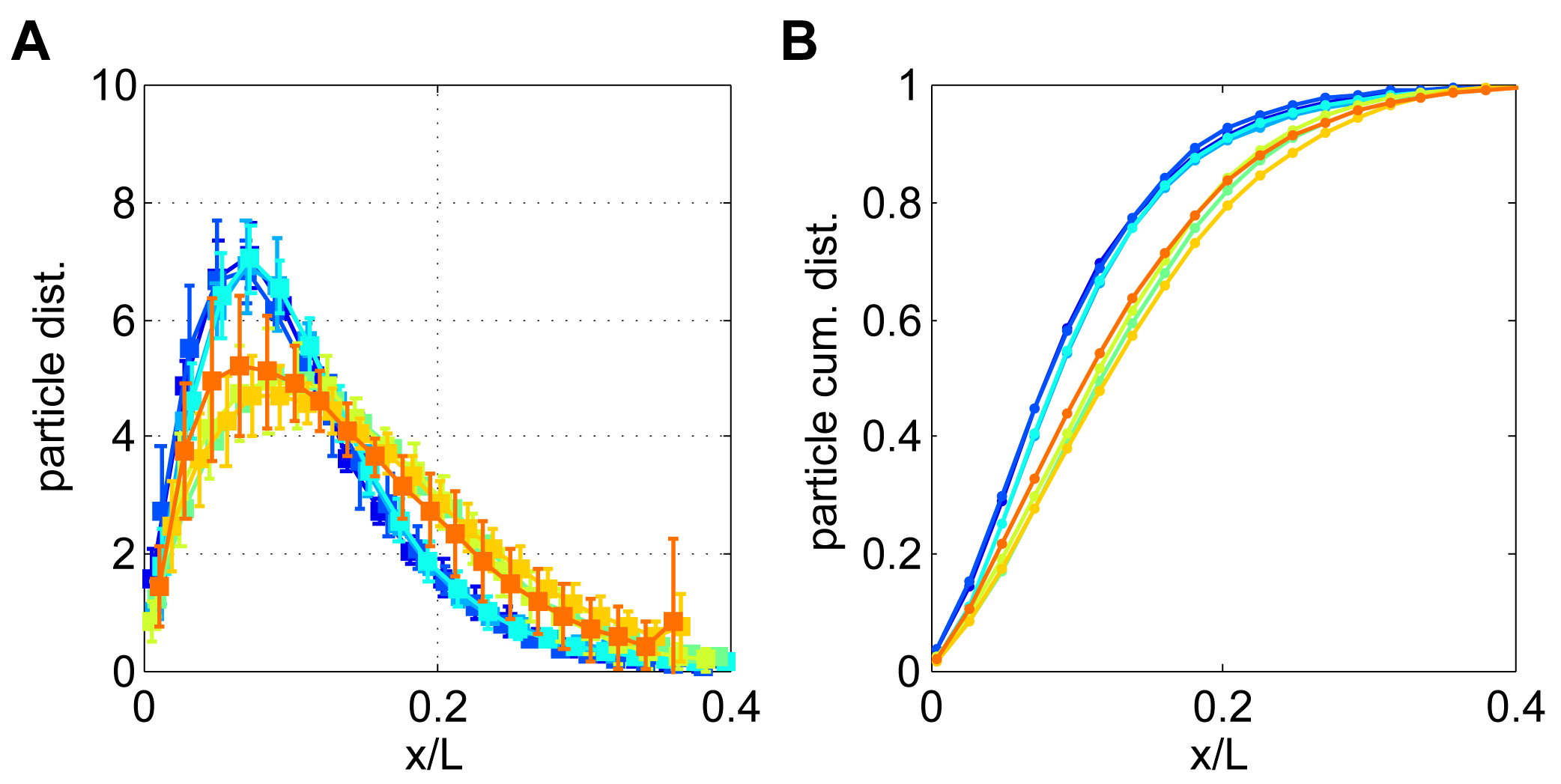

Supplement: Figure S6 — bcd mRNA particle number as a function of position along the AP axis. Shown are probability distributions of particle counts (A) and the cumulative distribution of particles (B) as a function of fractional distance along AP axis and nuclear cycle (color). The dataset and color code are equivalent to that shown in Figure 3. (0.23 MB TIF) [file pbio.1000596.s006.tif]

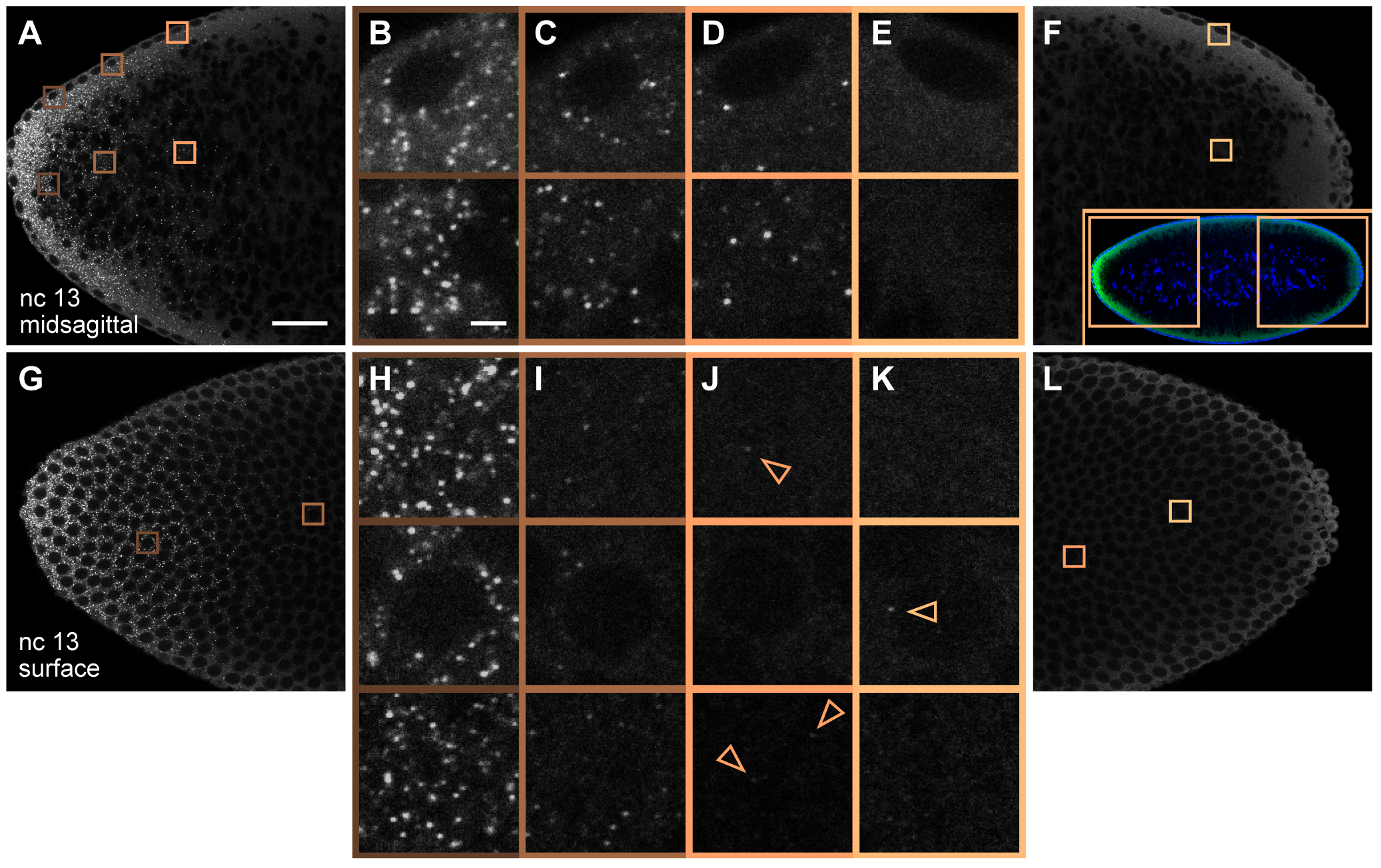

Supplement: Figure S7 — bcd mRNA particle distribution at n.c. 13. (A–F) n.c. 13 embryo near the midsagittal plane at the anterior (A) and posterior (F). Shaded boxes indicate magnified views (B–E) corresponding to cortex (upper) or core (lower). (F inset) Boxes indicate regions shown in (A) and (F). (G–L) Same embryo as (A–F), imaged at the nuclear layer. (H–K) Selected z-slices of boxed regions indicated in (G) and (L) showing apical (upper panels) and basal (lower panels) planes surrounding the nuclear layer (middle panels). Arrowheads indicate selected faint particles. Scale bars: 25 µm (A), 2 µm (B). (2.47 MB TIF) [file pbio.1000596.s007.tif]

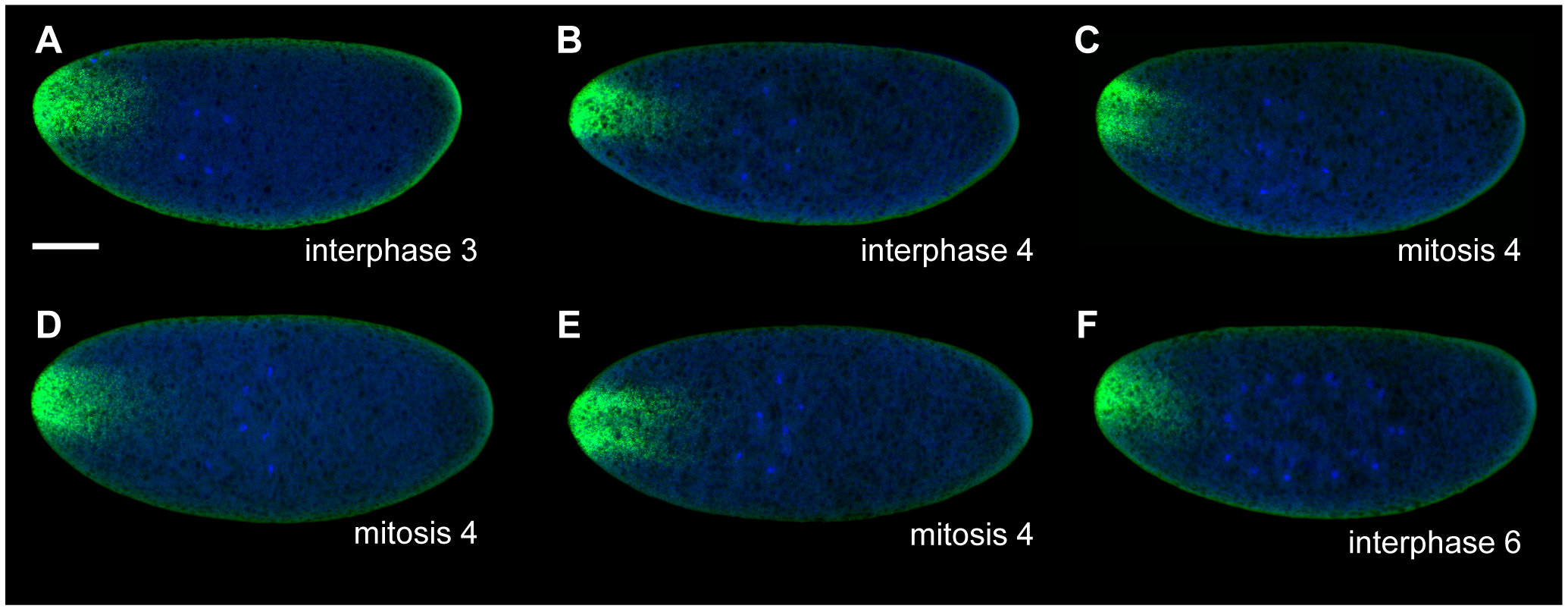

Supplement: Figure S8 — Low magnification FISH with multiple oligonucleotides in n.c. 3–6. Low magnification confocal images of midsagittal (A–C, F) or coronal (D–E) sections. Green, bcd mRNA; blue, DAPI. Scale bar: 50 µm. (1.49 MB TIF) [file pbio.1000596.s008.tif]

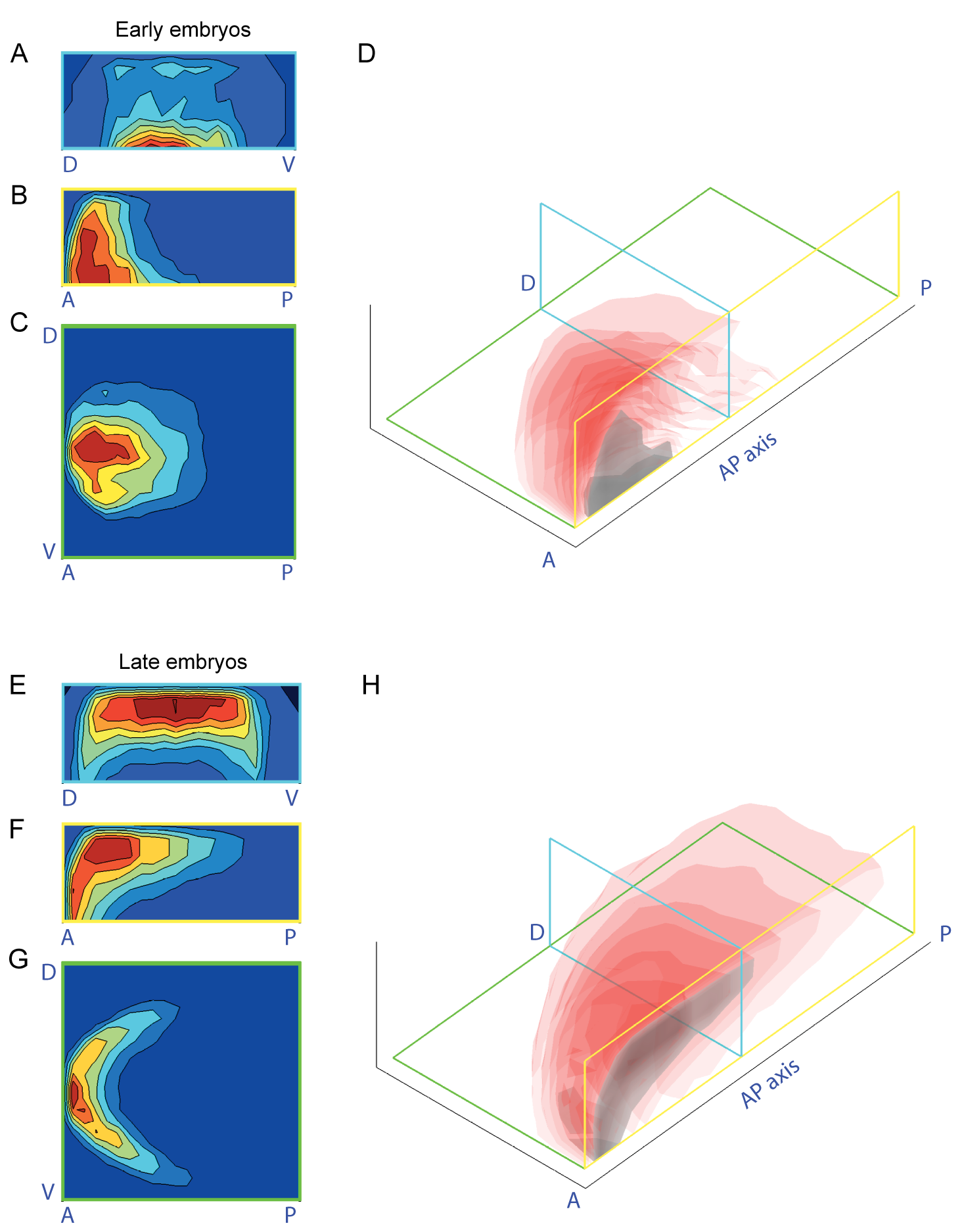

Supplement: Figure S9 — Schematic representation of bcd mRNA particle 3d distribution at early and late nuclear cycles. Locations of identified mRNA particles from early (until n.c. 6, A–D) and late (after n.c. 10, E–H) embryos were used to construct average 3d particle density profiles and typical cross-sections. (A–D) Sections of the transverse (blue frame, A), coronal (yellow frame, B), and midsagittal (green frame, C) planes of the 3d distribution (D) of early embryos show a wedge-shaped distribution of mRNA with a high concentration at the anterior pole A; redder color indicates higher particle concentrations. (E–H) In late embryos, bcd mRNA distribution resembles a cup covering the anterior pole, extending slightly farther into the posterior. Note the high concentration of particles in the cortex evident from the sections (E–G). (0.50 MB TIF) [file pbio.1000596.s009.tif]

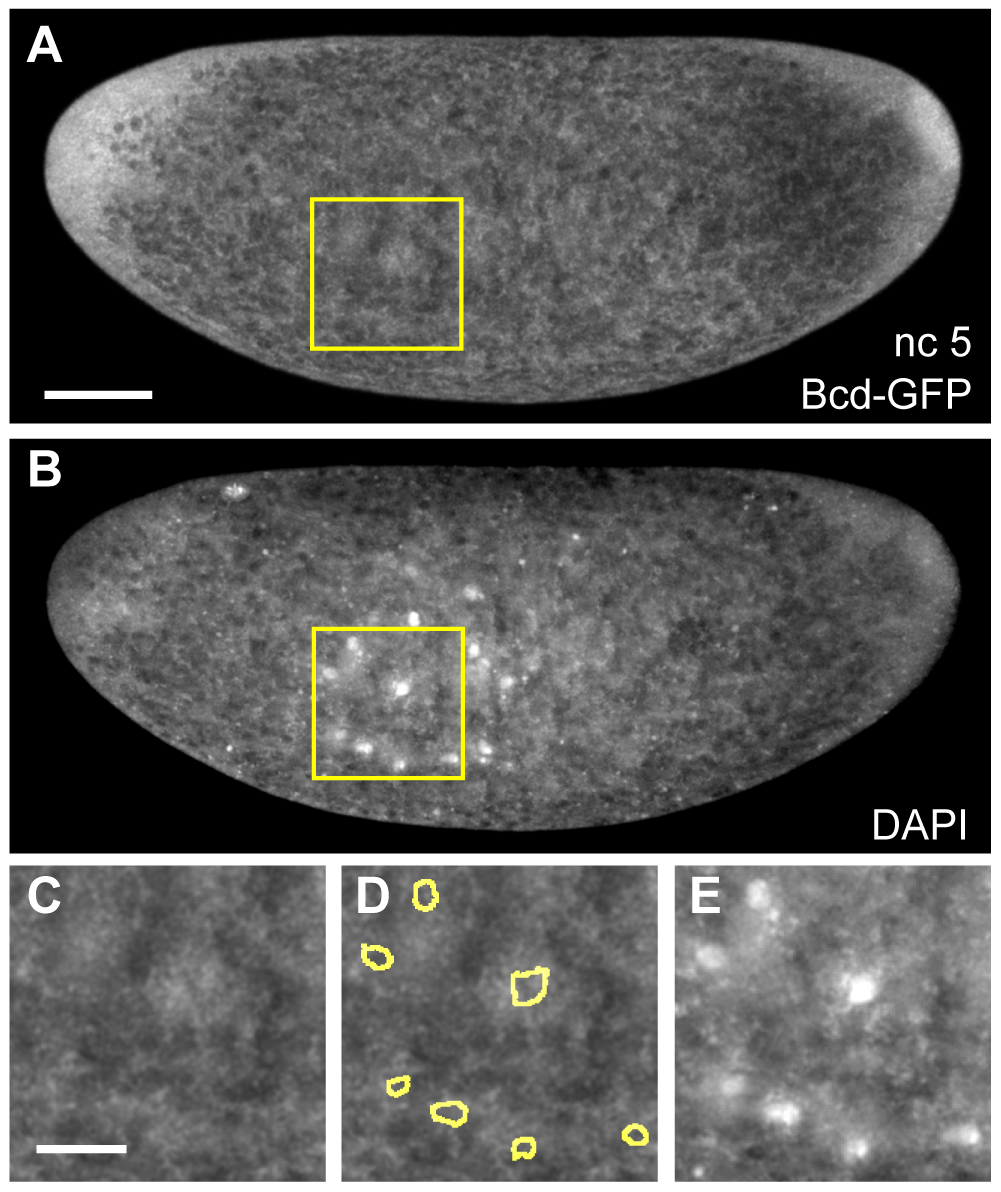

Supplement: Figure S10 — Nuclear accumulation of Bcd-GFP is not evident at interphase 5. Shown are maximum z-projections to display nuclei in multiple focal planes. (A) Bcd-GFP, (B) DAPI. Boxed regions in (A) and (B) are shown in (C) and (E), respectively. (D) Outlines of nuclear DAPI staining superimposed onto the image in (C). Scale bars: 50 µm (A), 20 µm (C). (0.88 MB TIF) [file pbio.1000596.s010.tif]

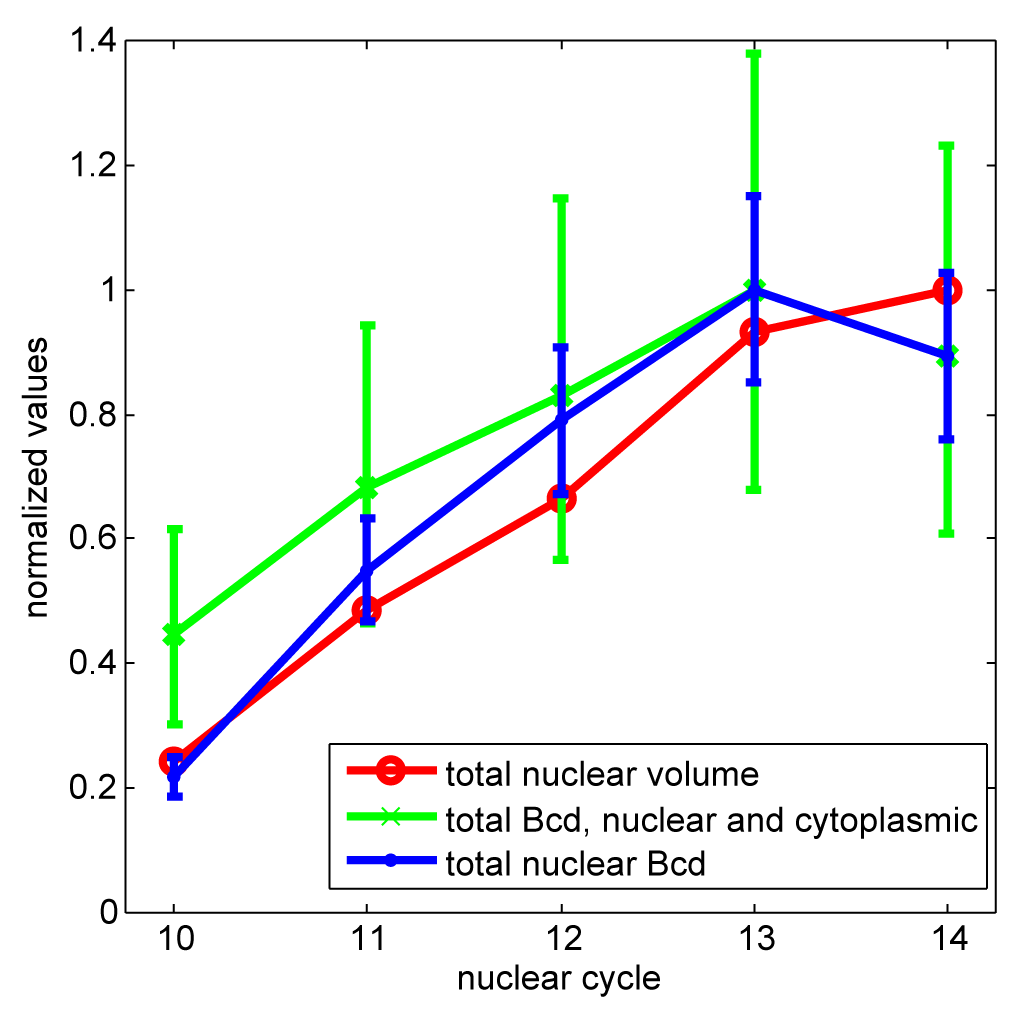

Supplement: Figure S11 — Estimated total Bcd during blastoderm stages. Nuclear volume (red curve) was calculated from previous measurements of nuclear diameter [9]. These values were multiplied by nuclear gradient amplitudes measured in this study (blue curve; error bars indicate measurement error of 15% observed in the anterior 50%), which were then divided by the fraction of Bcd localized to nuclei [9] to obtain total protein (green curve; error bars determined by applying 20% accuracy of nuclear Bcd estimates [9]). (0.10 MB TIF) [file pbio.1000596.s011.tif]

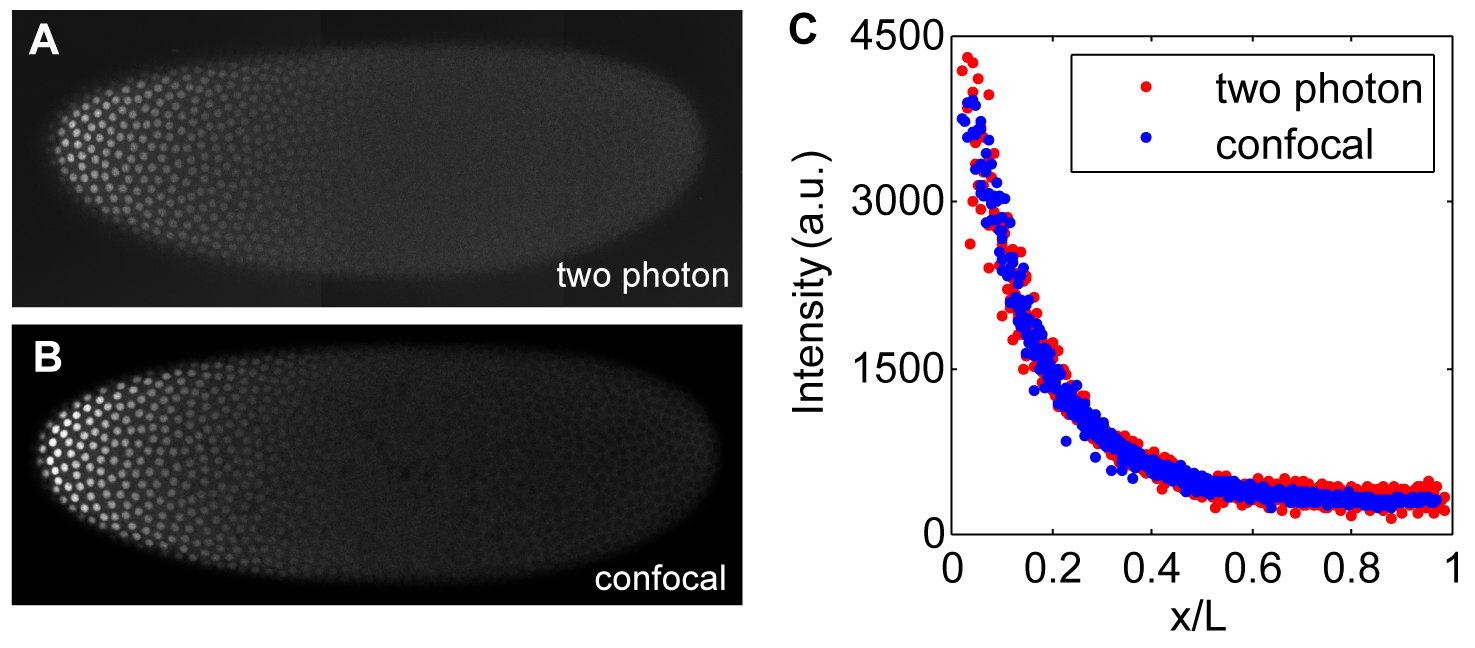

Supplement: Figure S12 — Confocal and two photon microscopy yield similar Bcd-GFP gradients in fixed embryos. (A) Example image obtained using custom built two-photon microscope as described [9] on the surface of Bcd-GFP autofluorescence in a fixed embryo at n.c. 13. (B) The same embryo as in (A), imaged by single photon confocal microscopy. (C) Nuclear gradients were extracted from each image. Raw intensity confocal values are shown. For comparison, values obtained by two photon microscopy were rescaled. (0.45 MB TIF) [file pbio.1000596.s012.tif]

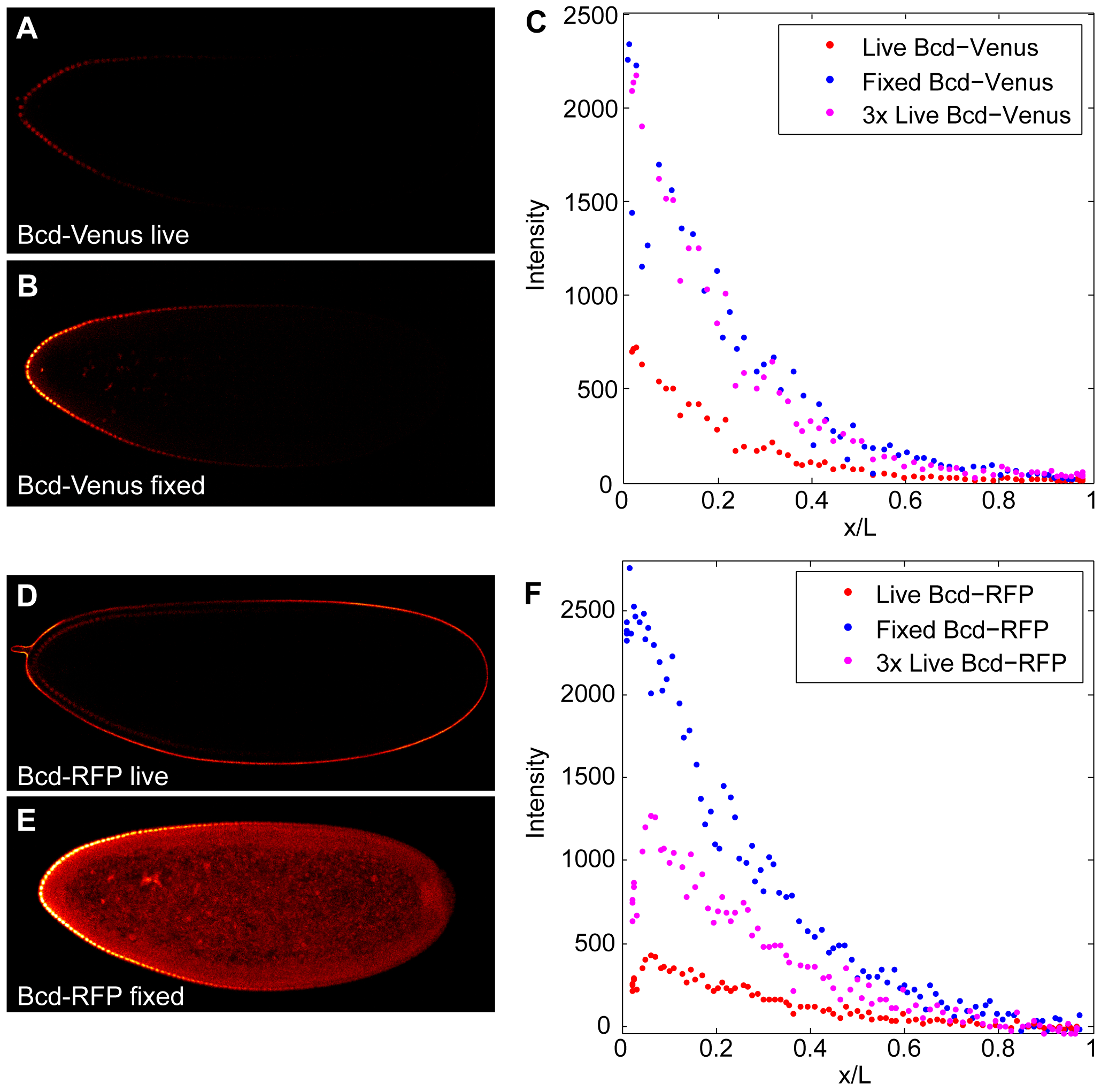

Supplement: Figure S13 — Bcd-Venus and Bcd-mRFP live and fixed gradients. Bcd-Venus [40] (A, B) and Bcd-RFP [40] (D, E) embryos were imaged live (A, D), fixed, re-imaged (B, E), and gradients extracted (C, F) as in Figure 9. In a live embryo, the vitelline membrane exhibits autofluoresence in the red channel and outlines the embryo (D); nuclei are observed within this outline. The membrane is removed prior to fixed imaging (E). (0.83 MB TIF) [file pbio.1000596.s013.tif]

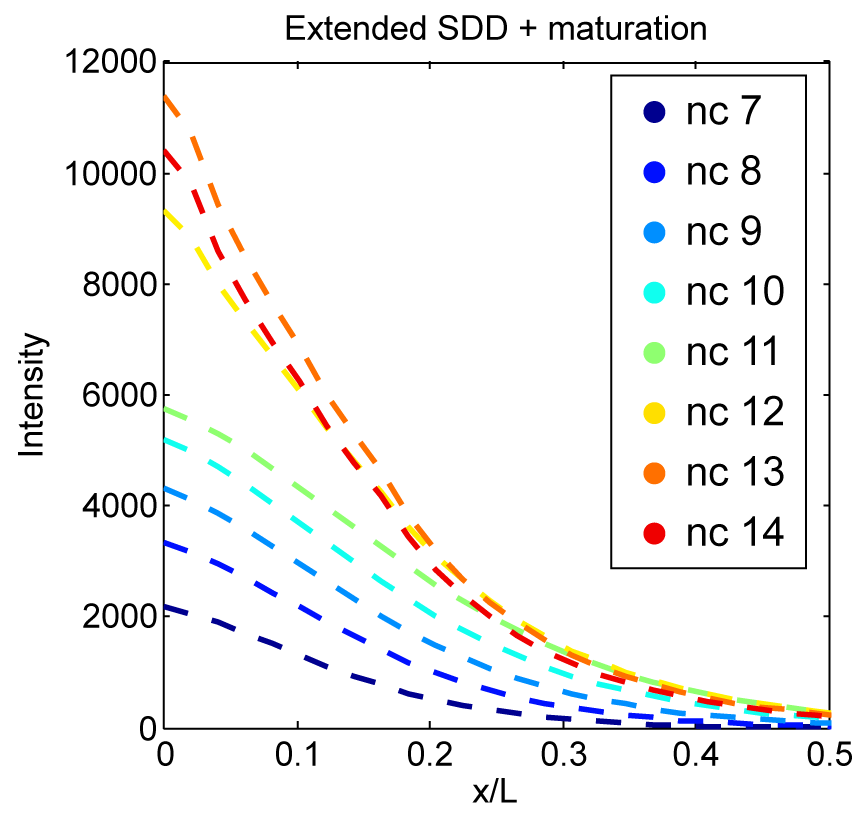

Supplement: Figure S14 — Simulated GFP maturation. The simulation of Figure 9B modified such that Bcd-GFP is not visible until 20 min after translation (see Materials and Methods). Other parameters are the same as in Figures 9 and S15. (0.10 MB TIF) [file pbio.1000596.s014.tif]

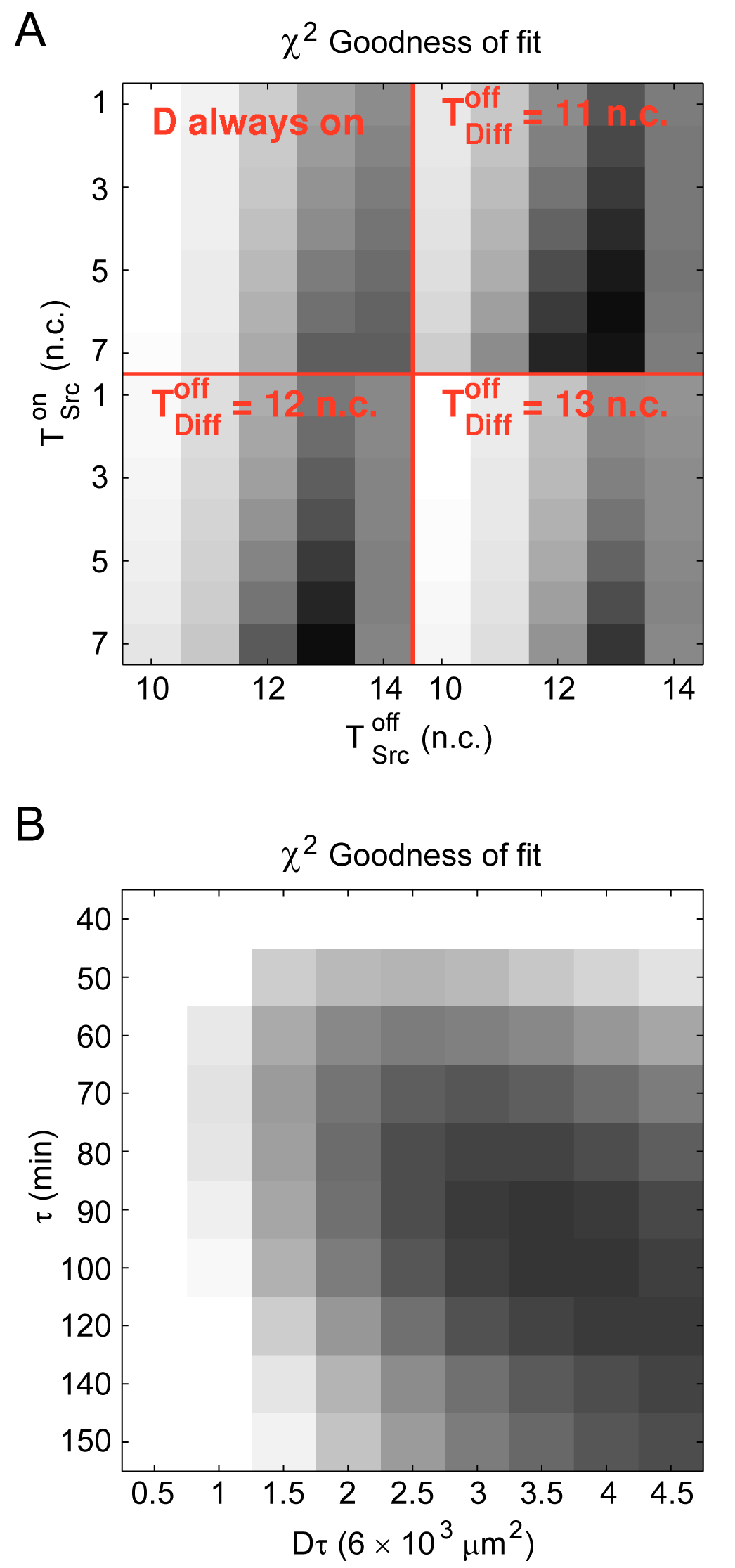

Supplement: Figure S15 — Exploration of parameter space for extended SDD modeling. Shown in grayscale are χ2–errors of fits of the model to measured mean Bcd protein gradients for n.c. 7–14; darker values correspond to smaller χ2 and better fits. (A) The starting and stopping times of bcd mRNA translation, TSrc off and TSrc on (x- and y-axes, respectively, in nuclear cycles), are varied for 4 scenarios, where diffusion is either always on or drops to zero in n.c. 11, 12, or 13 as denoted in each quadrant of (A). For each choice of these three timing parameters, we look for the best values for parameters D and τ. (B) D and τ search for TDiff off = 12, TSrc on = 7, TSrc off = 13. The best fits are obtained with D = 3.1 µm2/s and τ = 100 min; these values are used in Figure 9. (0.16 MB TIF) [file pbio.1000596.s015.tif]

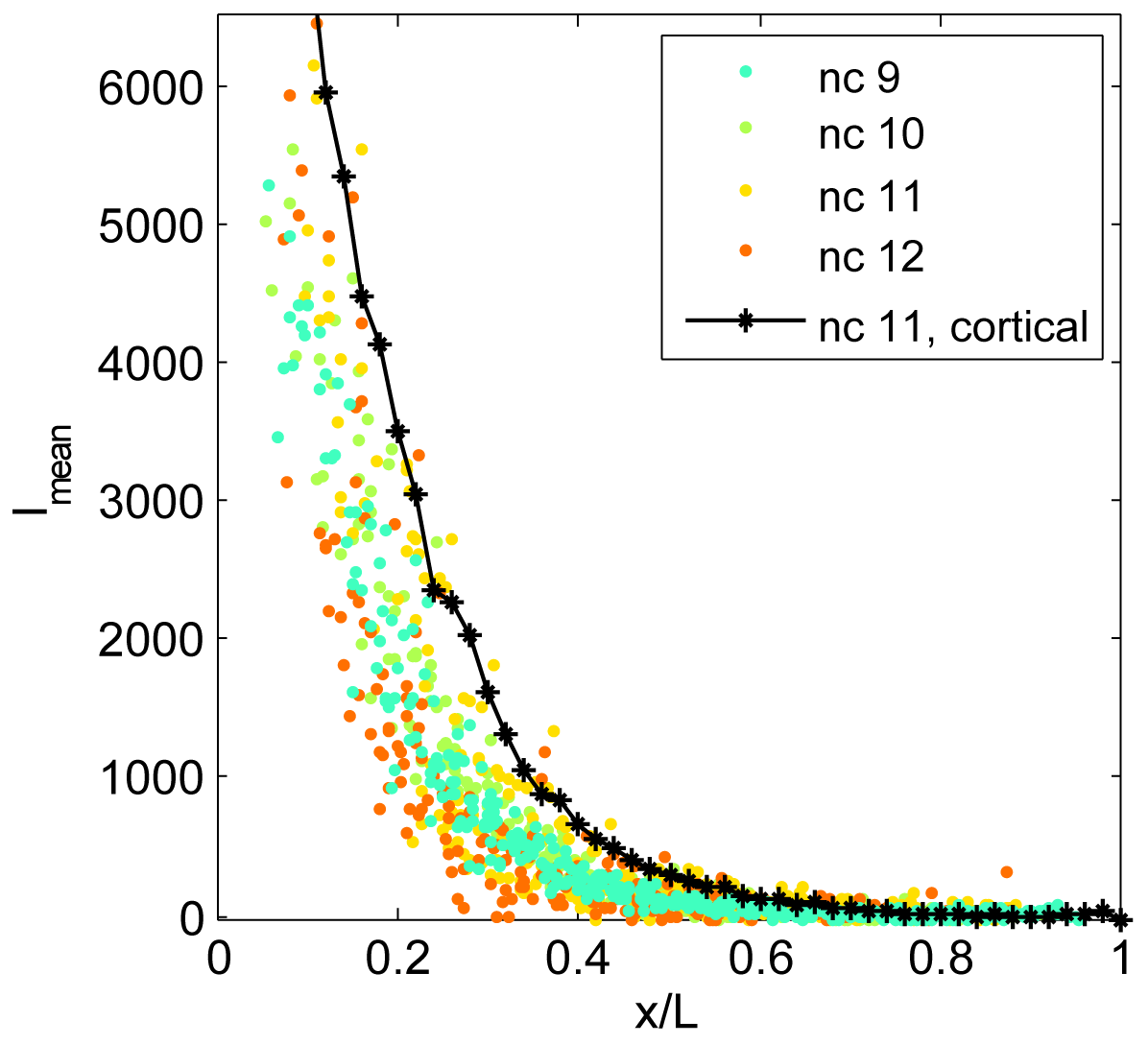

Supplement: Figure S16 — Bcd-GFP levels in yolk nuclei at blastoderm stages. Individual data points indicate mean attenuation-corrected intensities for individual nuclei, compared to binned means of dorsal cortical nuclei at n.c. 11 (black line). (0.15 MB TIF) [file pbio.1000596.s016.tif]
